# Association of Alk1 and Endoglin Polymorphisms with Cardiovascular Damage

**DOI:** 10.1038/s41598-020-66238-9

**Published:** 2020-06-10

**Authors:** Mercedes Garzon-Martinez, Nuria Perretta-Tejedor, Luis Garcia-Ortiz, Manuel A. Gomez-Marcos, Rogelio Gonzalez-Sarmiento, Francisco J. Lopez-Hernandez, Carlos Martinez-Salgado

**Affiliations:** 1 Institute of Biomedical Research of Salamanca (IBSAL), Salamanca, 37007 Spain; 2 Translational Research on Renal and Cardiovascular Diseases (TRECARD)-REDINREN (ISCIII), Department of Physiology and Pharmacology, University of Salamanca, Salamanca, 37007 Spain; 3 Primary Health Care Research Unit, La Alamedilla Health Centre, Health Service of Castilla y León (SACyL), Salamanca, 37003 Spain; 4 Department of Biomedical and Diagnostic Sciences, University of Salamanca, Salamanca, 37007 Spain; 5 Department of Medicine, University of Salamanca, Salamanca, 37007 Spain; 6 Institute of Molecular and Cellular Biology of Cancer (IBMCC), University of Salamanca-CSIC, Salamanca, 37007 Spain

**Keywords:** Cardiovascular biology, Cardiovascular diseases, Disease prevention, Prognosis

## Abstract

Cardiovascular diseases are associated to risk factors as obesity, hypertension and diabetes. The transforming growth factor-β1 receptors ALK1 and endoglin regulate blood pressure and vascular homeostasis. However, no studies relate the association of ALK1 and endoglin polymorphisms with cardiovascular risk factors. We analysed the predictive value of the ALK1 and endoglin polymorphisms on cardiovascular target organ damage in hypertensive and diabetic patients in 379 subjects with or without hypertension and diabetes in a Primary Care setting. The ALK1 rs2071219 polymorphism (AA genotype) is associated with a lower presence of diabetic retinopathy and with the absence of altered basal glycaemia. Being carrier of the ALK1 rs3847859 polymorphism (G allele) is associated with lower basal heart rate and with higher LDL-cholesterol levels. The endoglin rs3739817 polymorphism (AA genotype) is associated with higher levels of LDL-cholesterol, and being carrier of the endoglin rs10987759 polymorphism (C allele) is associated with higher haemoglobin levels and with an increased heart rate. Summarizing, several ALK1 and endoglin gene polymorphisms increase the risk of cardiovascular events. The analysis of these polymorphisms in populations at risk, in combination with the determination of other parameters and biomarkers, could implement the diagnosis and prognosis of susceptibility to cardiovascular damage.

## Introduction

Cardiovascular diseases are the main cause of death worldwide^1^, which are associated to common risk factors as obesity, hypertension (HT) and diabetes mellitus (DM)^2,3^. HT is the origin of cardiovascular complications such as peripheral arterial disease, ictus and heart attack. Cardiovascular risk increases markedly with high blood pressure (BP), DM and other risk factors^2^, including renal^4^, cardiac^5^ and vascular target organ damage^6,7^. Both large and small vessels may be affected in these syndromes. Atherosclerosis damages large vessels, whereas in disorders such as retinopathy, small vessels become altered. For instance, several studies have described the association of retinal vessel caliber with left ventricular hypertrophy (LVH)^8^, arterial HT^9^, metabolic syndrome^10^, cerebrovascular accident^11^, coronary diseases^12^ and cardiovascular risk.

Transforming growth factor-β1 (TGF-β1) participates in BP regulation and vascular homeostasis^13^. In recent years, our research group has studied the effect of several TGF-β1 receptors, such as ALK1 and endoglin, on cardiovascular and renal regulation. Endoglin (CD150, TGF III receptor) is involved in angiogenesis, and its role in cardiovascular risk is well documented^14^, as increased plasma levels of soluble endoglin are involved in coronary vasoconstriction, which may cause myocardial ischemia^15^, cardiac fibrosis and peripartum cardiomyopathy^16^. We have described how soluble endoglin levels are related with glycaemia, systolic BP, pulse pressure and presence of LVH^17^, and that endoglin is involved in endothelial regulation through cyclooxygenase-2 expression and activity^18^ and nitric oxide-dependent vasodilation^19^. Moreover, hypercholesterolemia, coronary heart disease, DM and HT contribute to increase serum endoglin levels^20^. Endoglin is also upregulated in experimental models of renal fibrosis^21^. On the other hand, ALK1 expression is self-regulated during periods of active angiogenesis^22^. Our group has also verified the regulatory role of ALK1 in arterial pressure and in cardiovascular physiopathology^23^ and in the development of renal fibrosis^24^.Despite the existence of these studies that relate ALK1 and endoglin receptors to vascular and renal pathophysiology and cardiovascular risk, at present there are no studies linking the presence of polymorphic variants in these genes with different cardiovascular risk factors. Therefore, we selected four polymorphisms of these two genes, ALK1 rs2071219 and rs3847859, and endoglin rs3739817 and rs10987759, all of them associated with vascular alterations (pulmonary hypertension, arteriovenous malformations), with high prevalence in the general population and whose presence apparently does not influence the biological activity of the protein, and we analyse the association and predictive value of these polymorphic variants on cardiovascular target organ damage in at-risk populations (HT and DM patients).

## Results

We included 379 subjects in the study. The distribution of genotypes of ALK1 rs2071219 and rs3847859 and endoglin rs3739817 and rs10987759 polymorphisms in control samples are in Hardy-Weinberg equilibrium (Table 1). Their demographic, clinical and physical variables are shown in Tables 2 and 3.

**Table 1:** Characteristics of the ALK1 and endoglin polymorphisms.

Gene	SNP ID	Base change	SNP	Chr location	Assay ID	HWE
ALK1	rs2071219	g.51913524 A > G	intron variant	12:51913524	C_15868502_10	>0,05
ALK1	rs3847859	g.51899716 G > A	intergenic	12:51899716	C_3240243_10	>0,05
ENG	rs3739817	c.1029 G > A	p.Thr343=	9:127824409	C_27491008_10	>0,05
ENG	rs10987759	g.127856098 T > C	utv	9:127856098	C_31370278_20	>0,05

Chr: chromosome; HWE: Hardy Weinberg equilibrium in control groups; SNP: single nucleotide polymorphism; utv: upstream transcript variant.

**Table 2:** Characteristics of the 379 patients included in the study.

	N	%
Male	201	53.0
Hypertension	277	73.1
Diabetes mellitus	88	23.2
Target organ damage	175	46.2
Altered basal glycaemia	45	11.9
BMI <25	67	17.7
BMI 25-30	197	52.0
BMI >30	115	30.3
Dyslipidaemia	259	68.3
Elevated PP	92	24.3
LVH	69	18.2
C-IMT	67	17.7
Altered PWV	58	15.3
PAD	6	1.6
CV risk <1%	53	14.0
CV risk 1–5%	181	47.8
CV risk 5–10%	78	20.6
CV risk >10%	67	17.7
Antihypertensive drugs	210	55.4
Antidiabetic drugs	61	16.1
Lipid-lowering drugs	149	39.3

BMI: body mass index; C-IMT: carotid intima media thickness; CV: cardiovascular; HT: hypertension; LVH: left ventricular hypertrophy; PAD: peripheral arterial disease; PP: pulse pressure; PWV: pulse wave velocity.

**Table 3:** Demographic, physical and basic analytical values of the patients included in the study.

	N	Average ± SD	Male		Female		P value
			N	Average ± SD	N	Average ± SD	
Weight. Kg	379	76.53 ± 14.70	201	82.95 ± 13.48	178	69.27 ± 12.51	> 0.05
Age. years	379	60.29 ± 9.70	201	59.35 ± 10.27	178	61.36 ± 8.93	> 0.05
BMI. Kg/m^2^	379	28.61 ± 4.42	201	28.91 ± 3.83	178	28.28 ± 4.99	> 0.05
SBP. mmHg	379	133.89 ± 17.19	201	136.62 ± 16.96	178	130.81 ± 16.97	> 0.05
DBP. mmHg	379	81.16 ± 10.56	201	82.76 ± 9.99	178	79.36 ± 10.91	> 0.05
PP. mmHg	379	52.73 ± 12.92	201	53.86 ± 13.50	178	51.45 ± 12.15	> 0.05
Heart rate. beats/min	379	69.74 ± 10.25	201	68.62 ± 10.95	178	71 ± 9.26	<0.05
ABI left	379	1.14 ± 0.10	201	1.15 ± 0.10	178	1.12 ± 0.09	> 0.05
ABI right	378	1.13 ± 0.10	201	1.15 ± 0.10	177	1.12 ± 0.09	> 0.05
Average C-IMT. mm	377	0.74 ± 0.10	199	0.75 ± 0.10	178	0.72 ± 0.09	> 0.05
Maximum C-IMT. mm	377	0.90 ± 0.12	199	0.92 ± 0.13	178	0.88 ± 0.11	> 0.05
PWV. m/s	374	8.65 ± 1.60	197	8.71 ± 1.67	177	8.57 ± 1.52	> 0.05
VDP-Cornell. mV/ms	372	1546.71 ± 561.74	201	1467.04 ± 622.79	171	1640.36 ± 464.75	<0.05
Basal glycaemia. mg/dL	379	95.46 ± 27.03	201	97.96 ± 30.51	178	92.64 ± 22.21	<0.05
Plasma creatinine. mg/dL	379	0.86 ± 0.19	201	0.95 ± 0.18	178	0.75 ± 0.14	<0.05
HDL-cholesterol. mg/dL	366	53.58 ± 14.09	196	54.33 ± 14.86	170	52.72 ± 13.14	> 0.05
LDL- cholesterol. mg/dL	373	132.76 ± 34.46	198	130.45 ± 35.53	175	135.37 ± 33.11	> 0.05
Triglycerides. mg/dL	379	128.05 ± 72.20	201	140.52 ± 83.03	178	113.96 ± 54.44	<0.05
HbA1c. %	370	5.92 ± 0.91	196	5.98 ± 0.99	174	5.85 ± 0.79	<0.05
Haemoglobin. g/dL	376	15.08 ± 1.15	200	15.57 ± 1.12	176	14.52 ± 0.89	<0.05
hs-CRP. mg/dL	360	0.31 ± 0.43	194	0.29 ± 0.39	166	0.33 ± 0.48	> 0.05
Fibrinogen. mg/dL	366	357.52 ± 65.32	196	347.59 ± 64.42	170	368.96 ± 64.66	> 0.05
Uric acid. mg/dL	379	5.26 ± 1.30	201	5.72 ± 1.14	178	4.73 ± 1.27	> 0.05
Urinary creatinine. mg/dL	372	104.38 ± 52.79	196	122.41 ± 53.23	176	84.30 ± 44.52	<0.05
Microalbuminuria. mg/dL	371	13.07 ± 66.78	195	21.21 ± 90.93	176	4.05 ± 10.33	<0.05
Leukocytes. *1000/μl	265	6.84 ± 1.77	143	7.15 ± 1.71	122	6.48 ± 1.77	> 0.05
Eosinophils. *1000/μl	265	0.21 ± 0.14	143	0.22 ± 0.15	122	0.19 ± 0.13	> 0.05
Lymphocytes. *1000/μl	265	2.49 ± 0.76	143	2.49 ± 0.76	122	2.41 ± 0.64	<0.05
Neutrophils. *1000/μl	265	3.53 ± 1.12	143	3.75 ± 1.08	122	3.28 ± 1.12	> 0.05
Left artery. μm	211	108.35 ± 13.61	112	106.85 ± 13.33	99	110.05 ± 13.77	> 0.05
Right artery. μm	212	108.93 ± 12.47	116	109.86 ± 12.02	96	107.81 ± 12.20	> 0.05
Average artery. μm	238	109.11 ± 12.07	129	108.90 ± 12.00	109	109.36 ± 12.20	> 0.05
Minor artery. μm	238	104.75 ± 12.09	129	104.83 ± 13.03	109	104.66 ± 13.21	> 0.05
Left vein. μm	211	140.93 ± 18.96	112	139.82 ± 18.15	211	140.93 ± 18.96	> 0.05
Right vein. μm	212	142.27 ± 19.15	116	142.99 ± 18.14	212	142.27 ± 19.15	> 0.05
Average vein. μm	238	142.05 ± 18.02	129	141.85 ± 15.94	99	142.18 ± 19.84	> 0.05
Major vein. μm	238	147.33 ± 18.95	129	147.68 ± 16.97	109	146.91 ± 21.12	> 0.05
Left AVIx. μm	176	0.78 ± 0.11	93	0.77 ± 0.11	83	0.79 ± 0.11	> 0.05
Right AVIx. μm	161	0.78 ± 0.11	86	0.78 ± 0.12	75	0.78 ± 0.10	> 0.05
Average AVIx. μm	236	0.78 ± 0.10	127	0.78 ± 0.10	109	0.78 ± 0.09	> 0.05

ABI: ankle brachial index; AVIx: arteriovenous index; BMI: body mass index; C- IMT: carotid intima media thickness; DBP: diastolic blood pressure; HbA1c: glycosylated hemoglobin; HDL: high-density lipoprotein; hs-CRP: high sensitive C-reactive protein; LDL: low-density lipoprotein; PP: pulse pressure; PWV: pulse wave velocity; SBP: systolic blood pressure; SD: standard deviation; VDP: voltage duration product. Artery, vein and AVIx values are retinal vessels.

The ALK1 rs2071219 polymorphism is related to the presence of early signs of diabetic retinopathy, as the presence of the AA genotype is associated with a higher mean AVIx, which would imply a lower presence of retinopathy in hypertensive and diabetic patients (Table 4). Moreover, being carrier of the A allele is associated with the absence of altered basal glycaemia (Table 4). Regarding the other polymorphism, there are significant differences in the ALK1 rs3847859 genotypic distribution between patients with basal heart rate <70 bmp (n = 219) or >70 bmp (n = 170). Being carrier of the G allele is associated with a lower basal heart rate in the recessive model (Tables 5 and 6). In addition, being carrier of the G allele is associated with higher LDL-cholesterol levels (Table 6).

**Table 4:** Distribution of ALK1 rs2071219 genotypes according to the medium caliber of retinal arterioles and to the presence of altered basal glycaemia.

SNP	Genotype	<average		>average		P value (1)	OR (95% CI) (1)	P value (2)	OR (95% CI) (2)
		N	%	N	%				
**Medium Caliber or Retinal Arterioles**									
rs2071219	GG	20	16.1	25	21.9	Ref.	1.000	Ref.	1.000
	AG	71	57.3	46	40.4	0.917	0.961 (0.457-2.024)	0.562	1.607 (0.323-7.993)
	AA	33	26.6	43	37.7	**0.017**	0.486 (0.269-0.877)	0.859	0.906 (0.306-2.686)
rs2071219 dominant	GG	20	16.1	25	21.9	Ref.	1.000	Ref.	1.000
	AG + AA	104	83.9	89	78.1	0.180	1.561 (0.814-2.994)	0.486	1.697 (0.384-7.508)
rs2071219 recessive	AA	33	26.6	43	37.7	Ref.	1.000	Ref.	1.000
	AG + GG	91	73.4	71	62.3	0.062	1.692 (0.974-2.938)	0.964	0.976 (0.348-2.741)
**SNP**	**Genotype**	**Yes**		**No**		**P value (1)**	**OR (95% CI) (1)**	**P value (2)**	**OR (95% CI) (2)**
		**N**	**%**	**N**	**%**				
**Presence of Altered Basal Glycaemia**									
rs2071219	GG	14	31.1	59	17.7	Ref.	1.000	Ref.	1.000
	AG	19	42.2	176	52.7	0.105	2.000 (0.864-4.629)	0.692	1.359 (0.298-6.207)
	AA	12	26.7	99	29.6	0.747	0.882 (0.410-1.895)	0.518	0.668 (0.197-2.268)
rs2071219 dominant	GG	14	31.1	59	17.7	Ref.	1.000	Ref.	1.000
	AG + AA	31	68.9	275	82.3	**0.030**	2.161 (1.079-4.329)	0.415	1.745 (0.457-6.668)
rs2071219 recessive	AA	12	26.7	99	29.6	Ref.	1.000	Ref.	1.000
	AG + GG	33	73.3	235	70.4	0.685	0.865 (0.428-1.746)	0.710	1.242 (0.396-3.889)

P value & OR adjusted by sex and age (1) and by sex, age, DM, HTA, DL, tobacco, alcohol and BMI (2). CI = confidence interval; OR = odd ratio; ref.=reference; SNP = single nucleotide polymorphism. Statistically significant results in bold.

**Table 5:** Distribution of ALK1 rs3847859 genotypes according to the basal heart rate.

SNP	Genotype	<70 bpm		>70 bpm		P value (1)	OR (95% CI) (1)	P value (2)	OR (95% CI) (2)
		N	%	N	%				
rs3847859	GG	77	36.0	43	26.1	Ref.	1.000	Ref.	1.000
	AG	115	53.7	88	53.3	**0.002**	0.362 (0.188-0.698)	**0.021**	0.272 (0.090-0.824)
	AA	22	10.3	34	20.6	**0.023**	0.495 (0.270-0.908)	0.051	0.366 (0.134-1.005)
rs3847859 dominant	GG	77	36.0	43	26.1	Ref.	1.000	Ref.	1.000
	AG + AA	137	64.0	122	73.9	**0.042**	0.628 (0.401-0.984)	0.200	0.625 (0.305-1.282)
rs3847859 recessive	AA	22	10.3	34	20.6	Ref.	1.000	Ref.	1.000
	AG + GG	192	89.7	131	79.4	**0.006**	2.263 (1.263-4.054)	**0.028**	3.005 (1.126-8.019)

P value & OR adjusted by sex and age (1) and by sex, age, DM, HTA, DL, tobacco, alcohol and BMI (2). Bpm: beats per minute; CI = confidence interval; OR = odd ratio; ref.=reference; SNP = single nucleotide polymorphism. Statistically significant results in bold.

**Table 6:** Statistically significant results in the distribution of ALK1 rs3847859 polymorphisms according to basal heart rate and plasma LDL cholesterol.

SNP		Heart rate	
		N	
**Basal Heart Rate**			
rs3847859	GG	120	68.5 ± 0.9
	AG	203	69.7 ± 0.7
	AA	56	72.6 ± 1.3
P value (1)	GG vs AG		0.877
	GG vs AA		0.035
	AG vs AA		0.170
P value (2)	GG vs AG		1.000
	GG vs AA		0.143
	AG vs AA		0.109
rs3847859 dominant	GG	120	68.5 ± 0.9
	AG + AA	259	71.2 ± 0.8
P value (1)			0.026
P value (2)			0.551
rs3847859 recessive	AA	56	72.6 ± 1.3
	AG + GG	323	69.1 ± 0.6
P value (1)			0.017
P value (2)			0.029
**SNP**		**LDL cholesterol**	
		**N**	
**LDL Cholesterol**			
rs3847859	GG	119	129.74 ± 3.11
	AG	199	137.02 ± 2.40
	AA	55	123.86 ± 4.56
P value (1)	GG vs AG		0.194
	GG vs AA		0.863
	AG vs AA		**0.033**
P value (2)	GG vs AG		1.000
	GG vs AA		1.000
	AG vs AA		1.000
rs3847859 dominant	GG	119	129.76 ± 3.11
	AG + AA	254	130.44 ± 2.58
P value (1)			0.861
P value (2)			0.764
rs3847859 recessive	AA	55	123.86 ± 4.56
	AG + GG	318	133.38 ± 1.96
P value (1)			0.056
P value (2)			0.522

P value adjusted by sex and age (1) and by sex, age, DM, HTA, DL, tobacco, alcohol and BMI (2). SD: standard deviation; SNP: single nucleotide polymorphism. Statistically significant results in bold.

The presence of the AA genotype in the endoglin rs3739817 polymorphism is associated with higher levels of LDL-cholesterol, as being carrier of the A allele is associated with higher levels of LDL-cholesterol in the dominant model (p = 0.055) (Table 7). Besides, being carrier of the C allele in the endoglin rs10987759 polymorphism is associated with higher haemoglobin levels in the dominant model (Table 8). In addition, being carrier of the C allele is associated with an increased heart rate also in the dominant model (Table 8).

**Table 7:** Statistically significant results in the distribution of endoglin rs3739817 polymorphisms according to plasma LDL cholesterol.

SNP		LDL Cholesterol	
		N	
rs3739817	GG	331	132.94 ± 1.87
	AG	40	128.68 ± 5.37
	AA	2	185.02 ± 24.01
P value (1)	GG vs AG		1.000
	GG vs AA		0.094
	AG vs AA		0.068
P value (2)	GG vs AG		1.000
	GG vs AA		0.246
	AG vs AA		0.281
rs3739817 dominant	GG	331	132.93 ± 1.87
	AG + AA	42	156.85 ± 12.30
P value (1)			0.055
P value (2)			0.642
rs3739817 recessive	AA	2	185.70 ± 24.01
	AG + GG	371	130.81 ± 2.84
P value (1)			**0.026**
P value (2)			0.081

P value adjusted by sex and age (1) and by sex, age, DM, HTA, DL, tobacco, alcohol and BMI (2). SNP: single nucleotide polymorphism. Statistically significant results in bold.

**Table 8:** Statistically significant results in the distribution of endoglin rs10987759 polymorphisms according to hemoglobin levels and heart rate.

SNP		Haemoglobin	
		N	
**Hemoglobin Levels**			
rs10987759	TT	319	15.02 ± 0.06
	CT	56	15.34 ± 0.14
	CC	1	17.17 ± 1.01
P value (1)	TT vs CT		0.101
	TT vs CC		0.104
	CT vs CC		0.220
P value (2)	TT vs CT		0.605
	TT vs CC		0.058
	CT vs CC		0.112
rs10987759 dominant	TT	319	15.02 ± 0.06
	CT + CC	57	16.26 ± 0.51
P value (1)			**0.017**
P value (2)			0.114
rs10987759 recessive	CC	1	17.17 ± 1.01
	CT + TT	375	15.18 ± 0.07
P value (1)			**0.050**
P value (2)			**0.022**
**SNP**		**Heart rate**	
		**N**	
**Heart Rate**			
rs10987759	TT	322	69.44 ± 0.56
	CT	56	71.12 ± 1.36
	CC	1	88.95 ± 10.14
P value (1)	TT vs CT		0.768
	TT vs CC		0.167
	CT vs CC		0.247
P value (2)	TT vs CT		1.000
	TT vs CC		0.219
	CT vs CC		0.297
rs10987759 dominant	TT	322	69.44 ± 0.56
	CT + CC	57	80.03 ± 5.11
P value (1)			**0.040**
P value (2)			0.357
rs10987759 recessive	CC	1	88.95± 10.14
	CT + TT	378	70.28 ± 0.73
P value (1)			0.067
P value (2)			0.077

P value adjusted by sex and age (1) and by sex, age, DM, HTA, DL, tobacco, alcohol and BMI (2). SNP: single nucleotide polymorphism. Statistically significant results in bold.

## Discussion

The relationship between different cardiovascular alterations and the presence of polymorphic variants in numerous genes has been documented extensively in recent years. Many of these studies have been conducted in populations of a specific ethnic origin, so their relevance has a local or geographical character, depending on the size and characteristics of the population included in the study. Several studies analyze the association of gene polymorphisms with cardiovascular disease in the Caucasian population^25–27^. These and many other studies show that the presence of polymorphic variants in numerous genes involved in cardiovascular regulation may favor the risk or predisposition to a broad spectrum of cardiovascular disorders.

However, although our research group and others have identified the involvement of TGF-β1 receptors in cardiovascular and renal damage (as described in the introduction section), to date there are no studies relating the presence of gene polymorphic variants in TGF-β1 receptors with cardiovascular damage. The only studies relating ALK1 and endoglin polymorphisms with cardiovascular damage show the absence of associations between the ALK1 rs2071219 polymorphism and the risks of brain arteriovenous malformations^28^, and between the endoglin rs3739817 polymorphism with human pulmonary arterial HP^29^. One study with the endoglin rs10987759 polymorphism shows a trend toward association with sporadic brain arteriovenous malformations, although it does not reach statistical significance^30^. Conversely, our study shows significant associations between ALK1 rs2071219, rs3847859 and endoglin rs3739817 and rs10987759 polymorphisms with several cardiovascular risk factors (retinopathy, altered basal glycaemia, heart rate, LDL-cholesterol, hemoglobin levels) in a Spanish population with or without HT and DM recruited in a primary care setting.

The association between reduction in the vascular calibre of the retinal vessels and cardiovascular risk is well known^31^. Patients with increased cardiovascular risk have more symptoms of retinopathy, such as dilated retinal veins and thinned arterioles. Both signs are associated with increased risk of stroke and coronary heart disease^32^. The thinning of the retinal arteries is related to the increase in pulse wave velocity and in pulse pressure^33^. TGF-β1 is involved in the thinning of the capillary basal lamina of the retina through its receptors ALK1 and ALK5 by upregulation of profibrotic genes in perycites^34^. The specific role of ALK1 in the vasculature is complex. In our study, being carrier of the A allele in the ALK1 rs2071219 polymorphism is associated with the absence of altered basal glycaemia, and the presence of the AA homozygous genotype is associated with a lower presence of retinopathy in HT and DM patients. Hyperglycaemia inhibits ALK1 expression, as shown *in vitro* in endothelial cells, or *in vivo* in streptozotocin-induced diabetes mellitus in mice^35^. ALK1 overexpression affects the migration and proliferation of human retinal capillary endothelial cells, thus promoting the remodelling of newly formed blood vessels and preventing angiogenesis^36^. The presence of the AA recessive genotype in the ALK1 rs2071219 polymorphism is associated with the absence of hyperglycaemia, and not elevated glucose levels prevents the inhibition of ALK1 expression, as previously described^35^. This normoglycaemic scenario with normal levels of ALK1 expression would result in a lower presence of retinopathy and would favour the migration and proliferation of endothelial cells and the remodelling of retinal blood vessels, although an in-depth mechanistic study would be needed to corroborate our hypothesis.

Heart rate variability is linked to cardiovascular risk factors such as HT and obesity, and decreased heart rate variability increases cardiovascular risk^37^. Moreover, patients in advanced chronic kidney disease stage have reduced heart rate variability^38^. Resting heart rate predicts cardiovascular diseases and longevity, and it is also an important marker of outcome in heart failure and other cardiovascular diseases^39^. High resting heart rate is also associated with increased risk of type 2 diabetes^40^. In our study, being carrier of the G allele in the ALK1 rs3847859 polymorphism is associated with a lower basal heart rate, which may be a genetic advantage in the face of the appearance of future cardiovascular complications, whereas being carrier of the C allele in the endoglin rs10987759 polymorphism is associated with an increased heart rate, circumstance that may increase the cardiovascular risk in these HT and DM patients. At this point in time, there is no study in the scientific literature describing the role of ALK1 and endoglin receptors in heart rate regulation, so we cannot explain how the presence of these genetic polymorphisms is associated to changes in heart rate. But our study opens a promising line of research that can assign a new role to these endothelial receptors in the regulation of heart rate.

LDL-cholesterol levels, even in those patients with normal values, are related to the presence and extent of systemic atherosclerosis, independently of other cardiovascular risk factors. As LDL-cholesterol levels increase there is a proportional increase in the prevalence of atherosclerosis and its thrombotic complications^41^. Reduction in LDL-cholesterol levels is beneficial to the reduction of atherosclerosis-related cardiovascular disease risk^42^. ALK1 expression is increased in human coronary atherosclerotic lesions^43^. In patients with hypercholesterolemia, ALK1 acts as a low-affinity, high-capacity receptor for LDL-cholesterol in endothelial cells. ALK1 binds LDL-cholesterol with lower affinity than the LDL-receptor and saturates only at hypercholesterolemic concentrations, and mediates LDL-cholesterol uptake in endothelial cells through an endocytic pathway that prevents the ligand from degradation and promotes LDL-cholesterol transcytosis, contributing to the initiation of atherosclerosis^44^. On the other hand, endoglin modulates ALK-1 ligand binding and signalling. Hypercholesterolemia alters endoglin expression and signalling, causing endothelial or vascular dysfunction before the initiation of atherosclerotic lesions^45^. All these findings suggest the participation of the endothelial receptors ALK1 and endoglin in the regulation of atherosclerosis, mainly exerting an antiatherogenic effect. Therefore, the identification of the presence of ALK1 rs3847859 and endoglin rs3739817 polymorphisms, which we have observed to be associated with higher LDL-cholesterol levels, could be of clinical relevance to identify patients with an increased atherosclerotic risk, and therefore, with a higher probability of suffering adverse cardiovascular events.

The relationship between total haemoglobin levels and cardiovascular risk is controversial. Increased haemoglobin concentration leads to increased blood viscosity, increased peripheral resistance and reduced blood flow and perfusion^46^. The Framingham Heart Study reported the relationship between haematocrit and cardiovascular disease incidence in women after adjusting for multiple cardiovascular risk factors^47^. Elevated haemoglobin levels are associated with acute myocardial infarction in men^48^. There is also an association between total haemoglobin levels and cardiovascular incidence (ischemic heart disease, stroke) in men^49^. Gender differences may be explained by the different haemoglobin concentration between men and women, whose levels might not be high enough to increase cardiovascular risk. However, the association found in our study is gender independent. On the other hand, the red blood cells membrane and the released haemoglobin have atherogenic activities, as extracellular and oxidized haemoglobin species induce lipid peroxidation and endothelial damage^50^. However, there are no studies directly relating endoglin to haemoglobin levels, although the regulatory role of this receptor in the processes of angiogenesis^51^ seems to suggest that it should have a role, although unknown, that may affect the haemoglobin levels in plasma. The fact that we have detected that being carrier of the C allele in the endoglin rs10987759 polymorphism is associated with higher haemoglobin levels reinforces this hypothesis.

One of the main limitations of our findings is that this is a retrospective study. Moreover, due to the number of analysed patients, the statistical power of the study is limited, so it is possible that some statistically significant differences that actually exist have not been detected. When interpreting these results, it should be taken into account that in our recruited population of hypertensive and diabetic patients there were not enough patients with endoglin rs10987759 CC homozygous genotype to obtain statistically valid conclusions, and thus we have only analysed the other two genotypes, CT and TT. Studies should be done in a larger population in order to confirm our results. We have not measured plasma soluble endoglin in all patients, because the phenotypic modifications caused by these polymorphisms are not detectable by the available antibodies or ELISAs for soluble endoglin. On the other hand, in preliminary studies we did not find differences in soluble endoglin plasma levels in patients with different polymorphisms of the endoglin gene, although the analysis was not performed in 100% of the recruited population. Moreover, it is also possible that these polymorphic variants do not affect the extracellular domain of endoglin (from which the soluble endoglin is released, after the proteolytic cleaving action of metalloproteinase MMP-14), but rather the structure of the membrane-bound endoglin.

Summarizing, ours study shows for the first time that the presence of certain ALK1 and endoglin polymorphic variants is associated to several cardiovascular risk factors (retinal artery thickness, altered basal glycaemia, heart rate, LDL-cholesterol and haemoglobin levels) in a Spanish population and therefore, the presence of these polymorphisms may be relevant to increase the risk of cardiovascular events in these patients. Our work reinforces the pre-existing knowledge of the influential role of these endothelial TGF-β1 receptors in cardiovascular regulation. Our findings suggest that the analysis of these polymorphisms in populations at risk (HT and DM patients), in combination with the determination of other parameters and biomarkers, could implement the diagnosis and prognosis of susceptibility to cardiovascular damage.

## Methods

This cross-sectional study was performed in 379 subjects aged 20-80 years with or without HT and DM. They were recruited in the Primary Care Research Unit of La Alamedilla Health Centre, Salamanca (Spain), covering a population of 46,000 inhabitants. We considered HT patients when the mean of three different BP measurements was ≥140 mm Hg for systolic blood pressure (SBP) or ≥ 90 mm Hg for diastolic blood pressure (DBP) or when patients received antihypertensive treatment. DM was diagnosed when basal plasma glucose ≥126 mg/dL, glycosylated hemoglobin (HbA1c) > 6,5% or when patients received antidiabetic treatment. Obesity was diagnosed by body mass index (BMI) ≥ 30 kg/m^2^. Dyslipidemia was diagnosed when total cholesterol >4.9 mmol/L (190 mg/dL) or low density lipoprotein cholesterol >3 mmol/L (115 mg/dL) or high-density lipoprotein cholesterol: men <1.0 mmol/L (40 mg/dL), women <1.2 mmol/L (46 mg/dL) or triglycerides >1.7 mmol/L (150 mg/dL)^52^. Exclusion criteria: patients recruited in a clinical trial or with serious comorbidities, and patients unable to follow the protocol requirement (psychological and/or cognitive disorders, failure to cooperate, educational limitations and problems in understanding written language, and failure to sign the informed consent document). Controls subjects (75 subjects, 51 men (68%), 24 women (32%)) were normotensive and normoglycaemic patients without detectable renal and cardiovascular alterations. We also evaluated previous history of cardiovascular disease, heart failure and cerebrovascular disease.

### Ethical and legal issues

The experimental protocol was in accordance with the Declaration of Helsinki (2008) of the World Medical Association, approved by the Institute of Biomedical Research of Salamanca (IBSAL) Ethics Committee and complied with Spanish data protection law (LO 15/1999) and specifications (RD 1720/2007). All participants recruited in the study signed an informed consent.

### Anthropometric measurements

We calculated BMI (kg/m^2^) measuring height with a portable system (Seca 222, Hamburg, Germany) and body weight using a homologated electronic scale (Seca 70; precision ± 0.1 kg).

### Plasma and urine determinations

We measured basal glucose, HbA1c, high-density lipoprotein (HDL)-cholesterol, low-density lipoprotein (LDL)-cholesterol, triglycerides, creatinine in plasma, and microalbumin and creatinine in urine in samples collected in the morning, as we previously described^7,17,53,54^, after fasting for at least 8 hours, using standard automatic techniques on a blind basis in the Biochemistry laboratory of the University Clinical Hospital, Salamanca (Spain).

### Blood pressure determination

We evaluate office BP after three measurements of SBP and DBP with a validated OMRON model M7 sphygmomanometer (Omron Health Care, Kyoto, Japan) following the recommendations of the European Society of Hypertension (ESH)^52^. We calculated SBP, DBP, and pulse pressure with the mean values of the second and third measurements.

### Evaluation of peripheral arterial disease

We analyzed the ankle–brachial index (ABI) at 22–24 °C in patients refrained from drinking coffee or smoking tobacco for at least 8 h before measurements. With patients lying in supine position resting for 20 min and with feet uncovered, we measured BP in the lower limbs with a portable Doppler system Minidop Es-100Vx (Hadeco Inc, Miyamae-ku Kawasaki, Japan). We calculated ABI for each foot by dividing the higher of the two SBP in the ankle by the higher of the two SBP in the arm. An ankle–brachial index <0.9 is considered pathological^52^.

### Determination of left ventricular hypertrophy

We performed electrocardiography (ECG) with a General Electric MAC 3.500 ECG System (Niskayuna, New York, USA) that measures wave voltage and duration and estimates Cornell voltage duration product (VDP)^55^. Left ventricular hypertrophy was defined as a Sokolow-Lyon index >3.5 mV; RaVL >1.1 mV, Cornell VDP > 244 mV*ms or RaVL >1.1 mV^52^.

### Determination of pulse wave velocity

We evaluated pulse wave velocity (PWV) with the SphygmoCor System (AtCor Medical Pty Ltd, Head Office, West Ryde, Australia) in the carotid and femoral arteries with patients in supine position measuring the delay with respect to the ECG wave. We obtained distance measurements with a measuring tape from the sternal notch to the carotid and femoral arteries at the sensor location.

### Assessment of carotid intima-media thickness

In order to optimize reproducibility, we obtained automated measurements of carotid intima-media thickness (IMT) with a Micromax ultrasound device (SonoSite Inc, Bothell, WA) paired with a 5–10 MHz multifrequency high-resolution linear transducer with Sonocal software. We made measurements of the common carotid in a 10 mm longitudinal section at 1 cm from the bifurcation in the proximal wall, and in the distal wall in the lateral, anterior and posterior projections, following an axis perpendicular to the artery in order to discriminate two lines: one for the intima-blood interface and the other for the media-adventitious interface. We obtained average values (average carotid IMT) and maximum values (maximum carotid IMT) automatically calculated by the software from six measurements of both the right and left carotid arteries)^56^. We considered abnormal average IMT if >0.90 mm or in the presence of atherosclerotic plaques with a diameter of 1.5 mm or a focal increase of 0.5 mm or 50% of the adjacent IMT^52^.

### Evaluation of renal function

We estimated glomerular filtration rate using the Chronic Kidney Disease Epidemiology Collaboration (CKD-EPI) equation^57^, the Modification of Diet in Renal Disease- Isotopic Dilution Mass Spectrometry (MDRD-IDMS)^58^ and proteinuria (albumin/creatinine ratio) following the criteria of the 2013 European Society of Hypertension/European Society of Cardiology Guidelines^52^. Subclinical renal damage is present when glomerular filtration rate is below 30–60 mL/min/1.73 m^2^ or microalbuminuria between 30–300 mg/24 h or albumin–creatinine ratio between 30–300 mg/g, 3.4–34 mg/mmol. We considered renal disease as a glomerular filtration rate <30 mL/min/1.73 m^2^, proteinuria > 300 mg/24 h or albumin/creatinine ratio> 300 mg/24 h^52^.

### Evaluation of retinopathy

We obtained nasal and temporal images centered on the disk using a Topcon TRC NW 200 non-mydriatic retinal camera (Topcon Europe B.C., Capelle a/d Ijssel, The Netherlands), as we previously described^17,54,59,60^. We loaded images into our own developed software, AV Index calculator (Registry no. 00/2011/589), which automatically estimates the mean caliber of veins and arteries as an arteriole-venule ratio, arteriovenous index (AVIx). An AVIx of 1.0 suggests that arteriolar and venular diameters in that eye are on average the same, whereas a smaller AVR suggests narrower arterioles. We used the pairs of main vessels in the upper and lower temporal quadrants, and we rejected all other vessels, in order to improve reliability and efficiency of the process, analyzing measures for each quadrant separately and together to estimate the mean measure in each eye.

### Cardiovascular risk assessment

We estimated morbidity and mortality cardiovascular risk (CVR) using the 2013 guidelines of the ESH^52^, based on cardiovascular risk factors, BP, asymptomatic organ damage and presence of diabetes, symptomatic cardiovascular disease or chronic kidney disease.

### DNA isolation and genotyping

We obtained genomic DNA from peripheral blood leukocytes by the phenol-chloroform method^61^. We identified ALK1 rs3847859 and rs2071219 and endoglin rs73739817 and rs10987759 polymorphisms using the allelic discrimination assay with TaqMan probes (Life Technologies, Carlsbad, California, USA) (Table 1), specific oligonucleotides to amplify the regions containing the polymorphisms and two labelled probes with the fluorochromes VIC and FAM to detect both alleles of each polymorphism^62^. We carried out the reaction with the Universal PCR Master Mix in the Real-Time PCR system of Step-One Plus (Applied Biosystems, Forster, CA, USA). A 5% of random samples were re-genotyped in order to ensure the reproducibility.

### Statistical analysis

We used the SPSS v.21.0 software (Armonk, New York, USA) as we previously described^63^. We used the chi-squared test for each polymorphism in order to test the conformity to the Hardy-Weinberg equilibrium in control group subjects. We analysed associations between the different clinical and molecular qualitative variables by cross tabs and the Pearson *X*^*2*^ test. We calculated the odds ratio (OR) and 95% confidence intervals with a logistic regression model to evaluate the association with the risk to develop the disease. We applied the ANOVA test to compare quantitative variables and the influence of polymorphism distribution in those cases that followed a parametric distribution (Levene’s test for homogeneity of variances, p > 0.05). We used the Mann Whitney U test when data followed a non-parametric distribution. We performed a statistical adjustment by sex and the continuous variable of age, and an additional statistical adjustment by HT, diabetes, dyslipidemia, abdominal obesity, tobacco and alcohol consumption, in order to consider confounding variables. The statistical power of the main hypothesis tests, accepting an alpha risk of 0.05 in a bilateral contrast with our sample of 379 subjects, is as follows: In the case of the rs2071219 dominant genotype and medium caliber of retinal arterioles, the statistical power to detect the difference between the mean of the first group (111.5 µm) and that of the second (108.5 µm) is 33%. In the case of the rs2071219 dominant genotype and altered basal glycemia, the statistical power to detect the difference between 19% of the first group and 10% of the second group is 57%, and in the case of the rs3847859 dominant genotype and the heart rate >70 bpm, the statistical power to detect the difference between 36% of the first group and 47% of the second is 55%. We considered statistically significant differences when P-value was <0.05. The statistical analysis of all correlations between the polymorphisms and the analysed variables that did not show statistically significant differences are shown in the supplementary tables.

## Supplementary information

Supplementary information.
