# Supplementary material for: Association of Alk1 and Endoglin Polymorphisms with Cardiovascular Damage
Source: Sci Rep. 2020 Jun 10;10:9383. doi: 10.1038/s41598-020-66238-9 (PMC7287057; doi:10.1038/s41598-020-66238-9)
Supplement: Supplementary file 1 — Supplementary information. [file 41598_2020_66238_MOESM1_ESM.pdf]

ASSOCIATION OF ALK1 AND ENDOGLIN POLYMORPHISMS WITH CARDIOVASCULAR DAMAGE

Mercedes Garzon-Martinez, Nuria Perretta-Tejedor, Luis Garcia-Ortiz, Manuel A. Gomez-Marcos, Rogelio Gonzalez-Sarmiento, Francisco J. Lopez-Hernandez, Carlos Martinez-Salgado.

SUPPLEMENTARY TABLES

P value & OR adjusted by sex and age. CI=confidence interval; OR=odd ratio; ref.=reference.

1. ALK1 rs2071219 polymorphism

MEDICAL RECORD

Table S1. Distribution of ALK1 rs2071219 genotypes according to the presence of previous personal history of cardiovascular disease.

SNP	Genotype	No		Yes		P value	OR (95% CI)
		N	%	N	%		
**rs2071219**	GG	71	19.6	2	11.8	Ref.	1.000
	AG	184	50.8	11	64.7	0.776	0.778 (0.138-4.385)
	AA	107	29.6	4	23.5	0.447	1.576 (0.488-5.092)
**rs2071219 dominant**	GG	71	19.6	2	11.8	Ref.	1.000
	AG+AA	291	80.4	15	88.2	0.368	0.494 (0.106-2.294)
**rs2071219 recessive**	AA	107	29.6	4	23.5	Ref.	1.000
	AG+GG	255	70.4	13	76.5	0.447	0.635 (0.196-2.051)

Table S2. Distribution of ALK1 rs2071219 genotypes according to the presence of previous familiar history of premature cardiovascular disease

SNP	Genotype	No		Yes		P value	OR (95% CI)
		N	%	N	%		
**rs2071219**	GG	71	19.6	2	11.8	Ref.	1.000
	AG	184	50.8	11	64.7	0.776	0.778 (0.138-4.385)
	AA	107	29.6	4	23.5	0.447	1.576 (0.488-5.092)
**rs2071219 dominant**	GG	71	19.6	2	11.8	Ref.	1.000
	AG+AA	291	80.4	15	88.2	0.368	0.494 (0.106-2.994)
**rs2071219 recessive**	AA	107	29.6	4	23.5	Ref.	1.000
	AG+GG	255	70.4	13	76.5	0.447	0.635 (0.196-2.051)

Table S3. Distribution of ALK1 rs2071219 genotypes according to the presence of previous personal history of cerebrovascular disease.

SNP	Genotype	No		Yes		P value	OR (95% CI)
		N	%	N	%		
**rs2071219**	GG	71	18.9	2	66.7	Ref.	1.000
	AG	195	51.8	0	0	0.417	2.757 (0.238-31.963)
	AA	110	29.3	1	33.3	0.995	0.000 (0.000-)
**rs2071219 dominant**	GG	71	18.9	2	66.7	Ref.	1.000
	AG+AA	305	81.1	1	33.3	0.093	8.078 (0.707-92.227)
**rs2071219 recessive**	AA	110	29.3	1	33.3	Ref.	1.000
	AG+GG	266	70.7	2	66.7	0.819	1.329 (0.117-15.103)

Table S4. Distribution of ALK1 rs2071219 genotypes according to the presence of previous personal history of heart failure.

SNP	Genotype	No		Yes		P value	OR (95% CI)
		N	%	N	%		
**rs2071219**	GG	72	19.5	1	10	Ref.	1.000
	AG	189	51.2	6	60	0.521	0.469 (0.047-4.721)
	AA	108	29.3	3	30	0.902	1.094 (0.263-4.554)
**rs2071219 dominant**	GG	72	19.5	1	10	Ref.	1.000
	AG+AA	297	80.5	9	90	0.442	0.429 (1.021-1.244)
**rs2071219 recessive**	AA	108	29.3	3	30	Ref.	1.000
	AG+GG	261	70.7	7	70	0.902	0.914 (0.220-3.807)

Table S5. Distribution of ALK1 rs2071219 genotypes according to the presence of previous personal history of dislipemia.

SNP	Genotype	No		Yes		P value	OR (95% CI)
		N	%	N	%		
**rs2071219**	GG	21	17.5	52	20.1	Ref.	1.000
	AG	62	51.7	133	51.3	0.477	1.268 (0.659-2.441)
	AA	37	30.8	74	28.6	0.866	1.045 (0.630-1.732)
**rs2071219 dominant**	GG	21	17.5	52	20.1	Ref.	1.000
	AG+AA	99	82.5	207	79.9	0.527	1.214 (0.665-2.217)
**rs2071219 recessive**	AA	37	30.8	74	28.6	Ref.	1.000
	AG+GG	83	69.2	185	71.4	0.866	0.957 (0.974-2.938)

Table S6. Distribution of ALK1 rs2071219 genotypes according to the presence of previous personal history of diabetes.

SNP	Genotype	No		Yes		P value	OR (95% CI)
		N	%	N	%		
**rs2071219**	GG	56	19.2	17	19.3	Ref.	1.000
	AG	151	51.9	44	50	0.822	0.922 (0.455-1.870)
	AA	84	28.9	27	30.7	0.666	0.885 (0.506-1.545)
**rs2071219 dominant**	GG	56	19.2	17	19.3	Ref.	1.000
	AG+AA	235	80.8	71	80.7	0.900	1.042 (0.544-1.997
**rs2071219 recessive**	AA	84	28.9	27	30.7	Ref.	1.000
	AG+GG	207	71.1	61	69.3	0.666	1.130 (0.647-1.975)

Table S7. Distribution of ALK1 rs2071219 genotypes according to the presence of previous personal history of hypertension.

SNP	Genotype	No		Yes		P value	OR (95% CI)
		N	%	N	%		
**rs2071219**	GG	24	23.5	49	17.7	Ref.	1.000
	AG	45	44.1	150	54.1	0.658	0.863 (0.449-1.657)
	AA	33	32.4	78	28.2	0.229	1.392 (0.812-2.384)
**rs2071219 dominant**	GG	24	23.5	49	17.7	Ref.	1.000
	AG+AA	78	76.5	228	82.3	0.123	0.620 (0.338-1.138)
**rs2071219 recessive**	AA	33	32.4	78	28.2	Ref.	1.000
	AG+GG	69	67.6	199	71.8	0.229	0.719 (0.419-1.231)

Table S8. Distribution of ALK1 rs2071219 genotypes according to the presence of total peripheral arterial disease.

SNP	Genotype	No		Yes		P value	OR (95% CI)
		N	%	N	%		
**rs2071219**	GG	65	18.7	1	20	Ref.	1.000

	AG	180	51.7	4	80	0.996	18413523.4 (0.000-)
	AA	103	29.6	0	0	0.996	27783572.9 (0.000-)
**rs2071219 dominant**	GG	65	18.7	1	20	Ref.	1.000
	AG+AA	283	81.3	4	80	0.719	0.663 (0.070-6.234)
**rs2071219 recessive**	AA	103	29.6	0	0	Ref.	1.000
	AG+GG	245	70.4	5	100	0.996	0.000 (0.000-)

Table S9. Distribution of ALK1 rs2071219 genotypes according to the presence of left ventricular hypertrophy (evaluated by electrocardiography)

SNP	Genotype	No		Yes		P value	OR (95% CI)
		N	%	N	%		
**rs2071219**	GG	68	19.9	4	13.3	Ref.	1.000
	AG	175	51.2	18	60.0	0610	0.724 (0.210-2.502)
	AA	99	28.9	8	26.7	0.572	1.285 (0.539-3.067)
**rs2071219 dominant**	GG	68	19.9	4	13.3	Ref.	1.000
	AG+AA	274	80.1	26	86.7	0.316	0.563 (0.184-1.728)
**rs2071219 recessive**	AA	99	28.9	8	26.7	Ref.	1.000
	AG+GG	243	71.1	22	73.3	0.572	0.778 (0.326-1.856)

Table S10. Distribution of ALK1 rs2071219 genotypes according to the presence of left ventricular hypertrophy (evaluated by Cornell voltage duration product)

SNP	Genotype	No		Yes		P value	OR (95% CI)
		N	%	N	%		
**rs2071219**	GG	68	19.5	4	17.4	Ref.	1.000
	AG	181	51.9	12	52.2	0.875	0.901 (0.247-3.289)
	AA	100	28.8	7	30.4	0.867	0.919 (0.343-2.464)
**rs2071219 dominant**	GG	68	19.5	4	17.4	Ref.	1.000
	AG+AA	281	80.5	19	82.6	0.973	0.989 (0.298-3.224)
**rs2071219 recessive**	AA	100	28.7	7	30.4	Ref.	1.000
	AG+GG	249	71.3	16	69.6	0.867	1.088 (0.406-2.915)

Table S11. Distribution of ALK1 rs2071219 genotypes according to the presence of left ventricular hypertrophy (evaluated by Sokolow-Lyon index)

SNP	Genotype	No		Yes		P value	OR (95% CI)
		N	%	N	%		
**rs2071219**	GG	83	19.6	0	0	Ref.	1.000
	AG	191	51.2	3	60	0.997	0.000 (0.000-.)
	AA	109	29.2	2	40	0.880	0.870 (0.142-5.334)
**rs2071219 dominant**	GG	73	19.6	0	0	Ref.	1.000
	AG+AA	300	80.4	5	100	0.997	0.000 (0.000-.)
**rs2071219 recessive**	AA	109	29.2	2	40	Ref.	1.000
	AG+GG	264	70.8	3	60	0.880	1.149 (0.187-7.045)

Table S12. Distribution of ALK1 rs2071219 genotypes according to the presence of left ventricular hypertrophy (evaluated by Lewis index)

SNP	Genotype	No		Yes		P value	OR (95% CI)
		N	%	N	%		
**rs2071219**	GG	40	17.3	6	17.6	Ref.	1.000
	AG	128	55.4	18	52.9	0.922	0.946 (0.314-2.852)
	AA	63	27.3	10	29.4	0.744	0.870 (0.376-2.010)
**rs2071219 dominant**	GG	40	17.3	6	17.6	Ref.	1.000
	AG+AA	191	82.7	28	82.4	0.869	1.088 (0.398-2.974)
	AA	63	27.3	10	29.4	Ref.	1.000

**rs2071219 recessive**	AG+GG	168	72.7	24	70.6	0.744	1.150 (0.498-2.658)

Table S13. Distribution of ALK1 rs2071219 genotypes according to the presence of abdominal obesity

SNP	Genotype	No		Yes		P value	OR (95% CI)
		N	%	N	%		
**rs2071219**	GG	36	20.5	37	18.2	Ref.	1.000
	AG	88	50.0	107	52.7	0.818	0.932 (0.511-1.700)
	AA	52	26.5	59	29.1	0.782	1.070 (0.665-1.721)
**rs2071219 dominant**	GG	36	20.5	37	18.2	Ref.	1.000
	AG+AA	140	79.5	166	81.8	0.622	0.871 (0.504-1.507)
**rs2071219 recessive**	AA	52	29.5	59	29.1	Ref.	1.000
	AG+GG	124	70.5	144	70.9	0.782	0.935 (0.581-1.504)

Table S14. Distribution of ALK1 rs2071219 genotypes according to the presence of metabolic syndrome

SNP	Genotype	No		Yes		P value	OR (95% CI)
		N	%	N	%		
**rs2071219**	GG	51	17.9	22	23.4	Ref.	1.000
	AG	149	52.3	46	48.9	0.362	1.377 (0.692-2.738)
	AA	85	29.8	26	27.7	0.932	1.025 (0.581-1.808)
**rs2071219 dominant**	GG	51	17.9	22	23.4	Ref.	1.000
	AG+AA	234	82.1	72	76.6	0.351	1.343 (0.723-2.495)
**rs2071219 recessive**	AA	85	29.8	26	27.7	Ref.	1.000
	AG+GG	200	70.2	68	72.3	0.932	0.975 (0.553-1.720)

ANALYTICAL VALUES

Table S15. Distribution of ALK1 rs2071219 genotypes according to the presence of elevated cholesterol plasma levels

SNP	Genotype	No		Yes		P value	OR (95% CI)
		N	%	N	%		
**rs2071219**	GG	18	15.8	54	21.4	Ref.	1.000
	AG	66	57.9	120	47.6	0.700	1.144 (0.577-2.271)
	AA	30	26.3	78	31.0	0.201	0.712 (0.423-1.199)
**rs2071219 dominant**	GG	18	15.8	54	21.4	Ref.	1.000
	AG+AA	96	84.2	198	78.6	0.132	1.608 (0.867-2.981)
**rs2071219 recessive**	AA	30	26.3	78	31.0	Ref.	1.000
	AG+GG	84	73.7	174	69.0	0.201	1.405 (0.834-2.367)

Table S16. Distribution of ALK1 rs2071219 genotypes according to the presence of reduced cholesterol-high density lipoproteins plasma levels

SNP	Genotype	No		Yes		P value	OR (95% CI)
		N	%	N	%		
**rs2071219**	GG	54	19.8	18	19.4	Ref.	1.000
	AG	142	52.0	44	47.3	0.679	0.863 (0.428-1.738)
	AA	77	28.2	31	33.3	0.356	0.770 (0.442-1.342)
**rs2071219 dominant**	GG	54	19.8	18	19.4	Ref.	1.000
	AG+AA	219	80.2	75	80.6	0.733	1.120 (0.814-2.153)
**rs2071219 recessive**	AA	77	28.2	31	33.3	Ref.	1.000
	AG+GG	196	71.8	62	66.7	0.356	1.299 (0.745-2.264)

Table S17. Distribution of ALK1 rs2071219 genotypes according to the presence of elevated cholesterol-low density lipoproteins plasma levels

SNP	Genotype	No		Yes		P value	OR (95% CI)
		N	%	N	%		
**rs2071219**	GG	25	21.9	48	18.5	Ref.	1.000
	AG	53	46.5	140	54.1	0.907	0.962 (0.506-1.830)
	AA	36	31.6	71	27.4	0.216	1.389 (0.825-2.339)
**rs2071219 dominant**	GG	25	21.9	48	18.5	Ref.	1.000
	AG+AA	89	78.1	211	81.5	0.223	0.693 (0.384-1.251)
**rs2071219 recessive**	AA	36	31.6	71	27.4	Ref.	1.000
	AG+GG	78	68.4	188	72.6	0.216	0.720 (0.428-1.212)

Table S18. Distribution of ALK1 rs2071219 genotypes according to the presence of elevated triglycerides plasma levels

SNP	Genotype	No		Yes		P value	OR (95% CI)
		N	%	N	%		
**rs2071219**	GG	49	17.3	24	25.0	Ref.	1.000
	AG	154	54.4	41	42.7	0.523	1.235 (0.646-2.365)
	AA	80	28.3	31	32.3	0.186	0.692 (0.401-1.194)
**rs2071219 dominant**	GG	49	17.3	24	25.0	Ref.	1.000
	AG+AA	234	82.7	72	75.0	0.060	1.786 (0.975-3.270)
**rs2071219 recessive**	AA	80	28.3	31	32.3	Ref.	1.000
	AG+GG	203	71.7	65	67.7	0.186	1.445 (0.838-2.494)

Table S19. Distribution of ALK1 rs2071219 genotypes according to the presence of dyslipemia

SNP	Genotype	No		Yes		P value	OR (95% CI)
		N	%	N	%		
**rs2071219**	GG	4	18.2	69	19.4	Ref.	1.000
	AG	13	59.1	181	50.8	0.835	0.863 (0.217-3.434)
	AA	5	22.7	106	29.8	0.485	0.680 (0.231-2.005)
**rs2071219 dominant**	GG	4	18.2	69	19.4	Ref.	1.000
	AG+AA	18	81.8	287	80.6	0.693	1.269 (0.388-4.151)
**rs2071219 recessive**	AA	5	22.7	106	29.8	Ref.	1.000
	AG+GG	17	77.3	250	70.2	0.485	1.470 (0.499-4.331)

Table S20. Distribution of ALK1 rs2071219 genotypes according to the presence of altered basal glycemia

SNP	Genotype	No		Yes		P value	OR (95% CI)
		N	%	N	%		
**rs2071219**	GG	59	17.7	14	31.1	Ref.	1.000
	AG	176	52.7	19	42.2	0.105	2.000 (0.864-4.629)
	AA	99	29.6	12	26.7	0.747	0.882 (0.410-1.895)
**rs2071219 dominant**	GG	59	17.7	14	31.1	Ref.	1.000
	AG+AA	275	82.3	31	68.9	0.030	2.161 (1.079-4.329)
**rs2071219 recessive**	AA	99	29.6	12	26.7	Ref.	1.000
	AG+GG	235	70.4	33	73.3	0.685	0.865 (0.428-1.746)

Table S21. Distribution of ALK1 rs2071219 genotypes according to the presence of microalbuminuria

SNP	Genotype	No		Yes		P value	OR (95% CI)
		N	%	N	%		
**rs2071219**	GG	68	19.3	4	20.0	Ref.	1.000
	AG	182	51.9	9	45.0	0.778	0.833 (0.243-2.968)
	AA	101	28.8	7	35.0	0.497	0.702 (0.253-1.949)
**rs2071219 dominant**	GG	68	19.4	4	20.0	Ref.	1.000
	AG+AA	283	80.6	16	80.0	0.783	1.186 (0.352-3.997)
**rs2071219 recessive**	AA	101	28.8	7	35.0	Ref.	1.000
	AG+GG	250	71.2	13	65.0	0.497	1.424 (0.513-3.951)

CARDIOVASCULAR TESTS

Table S22. Distribution of ALK1 rs2071219 genotypes according to the presence of elevated pulse pressure

SNP	Genotype	No		Yes		P value	OR (95% CI)
		N	%	N	%		
**rs2071219**	GG	13	14.1	60	20.9	Ref.	1.000
	AG	47	51.1	148	51.6	0.081	0.505 (0.235-1.087)
	AA	32	34.8	79	27.5	0.264	0.729 (0.418-1.270)
**rs2071219 dominant**	GG	13	14.1	60	20.9	Ref.	1.000
	AG+AA	79	85.9	227	79.1	0.316	0.693 (0.339-1.419)
**rs2071219 recessive**	AA	32	34.8	79	27.5	Ref.	1.000
	AG+GG	60	65.2	208	72.5	0.264	1.372 (0.788-2.391)

Table S23. Distribution of ALK1 rs2071219 genotypes according to the presence of increased carotid intima-media thickness

SNP	Genotype	No		Yes		P value	OR (95% CI)
		N	%	N	%		
**rs2071219**	GG	63	20.3	9	13.4	Ref.	1.000
	AG	157	50.6	37	55.2	0.213	0.577 (0.243-1.370)
	AA	90	29.1	21	31.4	0.947	0.980 (0.532-1.804)
**rs2071219 dominant**	GG	63	20.3	9	13.4	Ref.	1.000
	AG+AA	247	79.7	58	86.6	0.196	0.589 (0.264-1.314)
**rs2071219 recessive**	AA	90	29.0	21	31.3	Ref.	1.000
	AG+GG	220	71.0	46	68.7	0.947	1.021 (0.554-1.879)

Table S24. Distribution of ALK1 rs2071219 genotypes according to the values of pulse wave velocity

SNP	Genotype	< average		> average		P value	OR (95% CI)
		N	%	N	%		
**rs2071219**	GG	61	19.2	11	19.3	Ref.	1.000
	AG	163	51.5	29	50.9	0.973	1.017 (0.371-2.790)
	AA	93	29.3	17	29.8	0.997	0.998 (0.456-2.184)
**rs2071219 dominant**	GG	61	19.2	11	19.3	Ref.	1.000
	AG+AA	256	80.8	46	80.7	0.968	1.019 (0.403-2.994)
**rs2071219 recessive**	AA	93	29.3	17	29.8	Ref.	1.000
	AG+GG	224	70.7	40	70.2	0.997	1.002 (0.458-2.191)

Table S25. Distribution of ALK1 rs2071219 genotypes according to the presence of pathological ankle-brachial index

SNP	Genotype	< average		> average		P value	OR (95% CI)
		N	%	N	%		

**rs2071219**	GG	72	19.3	1	20.0	Ref.	1.000
	AG	191	51.2	4	80.0	0.996	15746068.6 (0.000-)
	AA	110	29.5	-	-	0.996	26096453.8 (0.000-)
**rs2071219 dominant**	GG	72	19.3	1	20.0	Ref.	1.000
	AG+AA	301	80.7	4	80.0	0.658	0.603 (0.064-5.656)
**rs2071219 recessive**	AA	110	29.5	-	-	Ref.	1.000
	AG+GG	263	70.5	5	100.0	0.996	0.000 (0.000-)

CURRENT DISEASES

Table S26. Distribution of ALK1 rs2071219 genotypes according to the presence of cerebrovascular disease

SNP	Genotype	No		Yes		P value	OR (95% CI)
		N	%	N	%		
**rs2071219**	GG	71	18.9	2	66.7	Ref.	1.000
	AG	195	51.8	-	-	0.417	2.757 (0.238-31.963)
	AA	110	29.3	1	33.3	0.995	0.000 (0.000-)
**rs2071219 dominant**	GG	71	18.9	2	66.7	Ref.	1.000
	AG+AA	305	81.1	1	33.3	0.093	8.078 (0.707-92.227)
**rs2071219 recessive**	AA	110	29.3	1	33.3	Ref.	1.000
	AG+GG	266	70.7	2	66.7	0.819	1.329 (0.117-15.103)

Table S27. Distribution of ALK1 rs2071219 genotypes according to the presence of cardiovascular disease

SNP	Genotype	No		Yes		P value	OR (95% CI)
		N	%	N	%		
**rs2071219**	GG	72	19.5	1	10.0	Ref.	1.000
	AG	189	51.2	6	60.0	0.521	0.469 (0.047-4.721)
	AA	108	29.3	3	30.0	0.902	1.094 (0.263-4.554)
**rs2071219 dominant**	GG	72	19.5	1	10.0	Ref.	1.000
	AG+AA	297	80.5	9	90.0	0.442	0.429 (0.220-3.708
**rs2071219 recessive**	AA	108	29.3	3	30.0	Ref.	1.000
	AG+GG	261	70.7	7	70.0	0.902	0.914 (0.220-3.807)

Table S28. Distribution of ALK1 rs2071219 genotypes according to the presence of kidney disease

SNP	Genotype	No		Yes		P value	OR (95% CI)
		N	%	N	%		
**rs2071219**	GG	70	19.1	2	50.0	Ref.	1.000
	AG	191	52.0	-	-	0.788	1.320 (0.174-9.997)
	AA	106	28.9	2	50.0	0.995	0.000 (0.000-)
**rs2071219 dominant**	GG	70	19.1	2	50.0	Ref.	1.000
	AG+AA	297	80.9	2	50.0	0.176	3.991 (0.538-29.600)
**rs2071219 recessive**	AA	106	28.9	2	50.0	Ref.	1.000
	AG+GG	261	71.1	2	50.0	0.316	2.783 (0.377-20.562)

Table S29. Distribution of ALK1 rs2071219 genotypes according to the presence of advanced retinopathy

SNP	Genotype	No		Yes		P value	OR (95% CI)
		N	%	N	%		
**rs2071219**	GG	25	19.4	1	12.5	Ref.	1.000
	AG	65	50.4	5	62.5	0.764	0.685 (0.058-8.097)

	AA	39	30.2	2	25.0	0.654	1.475 (0.270-8.069)
**rs2071219 dominant**	GG	25	19.4	1	12.5	Ref.	1.000
	AG+AA	104	80.6	7	87.5	0.497	0.465 (0.051-4.246)
**rs2071219 recessive**	AA	39	30.2	2	25.0	Ref.	1.000
	AG+GG	90	69.8	6	75.0	0.654	0.678 (0.124-3.708)

Table S30. Distribution of ALK1 rs2071219 genotypes according to the presence of controlled arterial pressure.

SNP	Genotype	No		Yesbvxb		P value	OR (95% CI)
		N	%	N	%		
**rs2071219**	GG	10	18.2	7	17.1	Ref.	1.000
	AG	30	54.5	19	46.3	0.440	0.616 (0.180-2.11)
	AA	15	27.3	15	36.6	0.286	0.600 (0.235-1.532)
**rs2071219 dominant**	GG	10	18.2	7	17.1	Ref.	1.000
	AG+AA	45	81.8	34	82.9	0.965	1.026 (0.326-3.255)
**rs2071219 recessive**	AA	15	27.3	15	36.6	Ref.	1.000
	AG+GG	40	72.7	26	63.4	0.286	1.666 (0.653-4.251)

Table S31. Distribution of ALK1 rs2071219 genotypes according to the presence of target organ damage

SNP	Genotype	< average		> average		P value	OR (95% CI)
		N	%	N	%		
**rs2071219**	GG	12	20.0	5	14.7	Ref.	1.000
	AG	31	51.7	17	50.0	0.378	0.559 (0.153-2.037)
	AA	17	28.3	12	35.3	0.568	0.758 (0.292-1.964)
**rs2071219 dominant**	GG	12	20.0	5	14.7	Ref.	1.000
	AG+AA	48	80.0	29	85.3	0.622	0.737 (0.220-2.474)
**rs2071219 recessive**	AA	17	28.3	12	35.3	Ref.	1.000
	AG+GG	43	71.7	22	64.7	0.568	1.320 (0.509-3.422)

Table S32. Distribution of ALK1 rs2071219 genotypes according to the presence of hypertension, diabetes and obesity

SNP	Genotype	No		Yes		P value	OR (95% CI)
		N	%	N	%		
**rs2071219**	GG	24	19.8	49	19.0	Ref.	1.000
	AG	61	50.4	134	51.9	0.921	0.968 (0.513-1.828)
	AA	36	29.8	75	29.1	0.860	1.046 (0.632-1.732)
**rs2071219 dominant**	GG	24	19.8	49	19.0	Ref.	1.000
	AG+AA	97	80.2	209	81.0	0.793	0.925 (0.518-1.654)
**rs2071219 recessive**	AA	36	29.8	75	29.1	Ref.	1.000
	AG+GG	85	70.2	183	70.9	0.860	0.956 (0.577-1.582)

Table S33. Distribution of ALK1 rs2071219 genotypes according to the presence of hypertension and diabetes

SNP	Genotype	No		Yes		P value	OR (95% CI)
		N	%	N	%		
**rs2071219**	GG	33	20.9	40	18.1	Ref.	1.000
	AG	74	46.8	121	54.8	0.964	1.014 (0.554-1.855)
	AA	51	32.3	60	27.1	0.181	1.388 (0.859-2.244)
**rs2071219 dominant**	GG	33	20.9	40	18.1	Ref.	1.000
	AG+AA	125	79.1	181	81.9	0.267	0.730 (0.419-1.272)
	AA	51	32.3	60	27.1	Ref.	1.000

**rs2071219 recessive**	AG+GG	107	67.7	161	72.9	0.181	0.720 (0.446-1.164)

RISK SCALES

Table S34. Distribution of ALK1 rs2071219 genotypes according to the presence of cardiovascular risk

SNP	Genotype	< average		> average		P value	OR (95% CI)
		N	%	N	%		
**rs2071219**	GG	6	19.4	66	19.7	Ref.	1.000
	AG	14	45.1	172	51.3	0.555	1.424 (0.440-4.603)
	AA	11	35.5	97	29.0	0.565	1.315 (0.518-3.339)
**rs2071219 dominant**	GG	6	19.4	66	19.7	Ref.	1.000
	AG+AA	25	80.6	269	80.3	0.890	1.083 (0.349-3.364)
**rs2071219 recessive**	AA	11	35.5	97	29.0	Ref.	1.000
	AG+GG	20	64.5	238	71.0	0.565	0.761 (0.299-1.931)

Table S35. Distribution of ALK1 rs2071219 genotypes according to the presence of diabetes (based on 2013 guidelines)

SNP	Genotype	No		Yes		P value	OR (95% CI)
		N	%	N	%		
**rs2071219**	GG	62	19.6	11	17.5	Ref.	1.000
	AG	163	51.6	32	50.8	0.552	0.780 (0.345-1.765)
	AA	91	28.8	20	31.7	0.662	0.870 (0.466-1.625)
**rs2071219 dominant**	GG	62	19.6	11	17.5	Ref.	1.000
	AG+AA	254	80.4	52	82.5	0.778	0.897 (0.421-1.911)
**rs2071219 recessive**	AA	91	28.8	20	31.7	Ref.	1.000
	AG+GG	225	71.2	43	68.3	0.662	1.149 (0.616-2.146)

Table S36. Distribution of ALK1 rs2071219 genotypes according to the presence of hypertension (based on 2013 guidelines)

SNP	Genotype	No		Yes		P value	OR (95% CI)
		N	%	N	%		
**rs2071219**	GG	39	20.0	34	18.5	Ref.	1.000
	AG	95	48.7	100	54.3	0.864	1.054 (0.579-1.917)
	AA	61	31.3	50	27.2	0.312	1.276 (0.796-2.045)
**rs2071219 dominant**	GG	39	20.0	34	18.5	Ref.	1.000
	AG+AA	156	80.0	150	81.5	0.492	0.826 (0.479-1.424)
**rs2071219 recessive**	AA	61	31.3	50	27.2	Ref.	1.000
	AG+GG	134	68.7	134	72.8	0.312	0.784 (0.489-1.257)

RETINOPATHY

Table S37. Distribution of ALK1 rs2071219 genotypes according to the caliber of medium retinal arteries

SNP	Genotype	< average		> average		P value	OR (95% CI)
		N	%	N	%		
**rs2071219**	GG	20	16.1	25	21.9	Ref.	1.000
	AG	71	57.3	46	40.4	0.917	0.961 (0.457-2.024)
	AA	33	26.6	43	37.7	0.017	0.486 (0.269-0.877)
	GG	20	16.1	25	21.9	Ref.	1.000

**rs2071219 dominant**	AG+AA	104	83.9	89	78.1	0.180	1.561 (0.814-2.994)
**rs2071219 recessive**	AA	33	26.6	43	37.7	Ref.	1.000
	AG+GG	91	73.4	71	62.3	0.062	1.692 (0.974-2.938)

Table S38. Distribution of ALK1 rs2071219 genotypes according to the caliber of right retinal arteries

**SNP**	**Genotype**	**< average**		**> average**		**P value**	**OR (95% CI)**
		N	%	N	%		
**rs2071219**	GG	18	16.7	22	21.2	Ref.	1.000
	AG	56	51.9	46	44.2	0.730	1.148 (0.525-2.507)
	AA	34	31.5	36	34.6	0.469	0.797 (0.430-1.475)
**rs2071219 dominant**	GG	18	16.7	22	21.2	Ref.	1.000
	AG+AA	90	83.3	82	78.8	0.335	1.441 (0.686-3.025)
**rs2071219 recessive**	AA	34	31.5	36	34.6	Ref.	1.000
	AG+GG	74	68.5	68	65.4	0.469	1.255 (0.678-2.324)

Table S39. Distribution of ALK1 rs2071219 genotypes according to the caliber of minor retinal arteries

**SNP**	**Genotype**	**< average**		**> average**		**P value**	**OR (95% CI)**
		N	%	N	%		
**rs2071219**	GG	17	13.8	28	24.3	Ref.	1.000
	AG	70	56.9	47	40.9	0.305	1.484 (0.698-3.158)
	AA	36	29.3	40	34.8	0.110	0.620 (0.345-1.114)
**rs2071219 dominant**	GG	17	13.8	28	24.3	Ref.	1.000
	AG+AA	106	86.2	87	75.7	0.016	2.392 (1.176-4.865)
**rs2071219 recessive**	AA	36	29.3	40	34.8	Ref.	1.000
	AG+GG	87	70.7	75	65.2	0.110	1.612 (0.897-2.895)

Table S40. Distribution of ALK1 rs2071219 genotypes according to the caliber of left retinal arteries

**SNP**	**Genotype**	**< average**		**> average**		**P value**	**OR (95% CI)**
		N	%	N	%		
**rs2071219**	GG	17	16.3	26	24.3	Ref.	1.000
	AG	59	56.7	44	41.1	0.676	1.184 (0.535-2.620)
	AA	28	26.9	37	34.6	0.066	0.551 (0.292-1.041)
**rs2071219 dominant**	GG	17	16.3	26	24.3	Ref.	1.000
	AG+AA	87	83.7	81	75.7	0.042	2.150 (1.028-4.497)
**rs2071219 recessive**	AA	28	26.9	37	34.6	Ref.	1.000
	AG+GG	76	73.1	70	65.4	0.066	1.815 (0.960-3.430)

Table S41. Distribution of ALK1 rs2071219 genotypes according to the values of left arteriovenous index (AVIx)

**SNP**	**Genotype**	**< average**		**> average**		**P value**	**OR (95% CI)**
		N	%	N	%		
**rs2071219**	GG	17	20.2	19	20.7	Ref.	1.000
	AG	37	44.1	46	50.0	0.570	1.278 (0.549-2.976)
	AA	30	35.7	27	29.3	0.334	1.402 (0.706-2.782)
**rs2071219 dominant**	GG	17	20.2	19	20.7	Ref.	1.000
	AG+AA	67	79.8	73	79.3	0.820	0.912 (0.411-2.020)
**rs2071219 recessive**	AA	30	35.7	27	29.3	Ref.	1.000
	AG+GG	54	64.3	65	70.7	0.334	0.713 (0.359-1.416)

Table S42. Distribution of ALK1 rs2071219 genotypes according to the values of right arteriovenous index (AVIx)

SNP	Genotype	< average		> average		P value	OR (95% CI)
		N	%	N	%		
**rs2071219**	GG	18	22.0	14	17.7	Ref.	1.000
	AG	36	43.9	39	49.4	0.697	0.840 (0.348-2.026)
	AA	28	43.1	26	32.9	0.720	1.137 (0.562-2.301)
**rs2071219 dominant**	GG	18	22.0	14	17.7	Ref.	1.000
	AG+AA	64	78.0	65	82.3	0.477	0.738 (0.320-1.705)
**rs2071219 recessive**	AA	28	34.1	26	32.9	Ref.	1.000
	AG+GG	54	65.9	53	67.1	0.720	0.879 (0.435-1.779)

Table S43. Distribution of ALK1 rs2071219 genotypes according to the values of medium arteriovenous index (AVIx)

SNP	Genotype	< average		> average		P value	OR (95% CI)
		N	%	N	%		
**rs2071219**	GG	28	22.8	16	14.2	Ref.	1.000
	AG	54	43.9	63	55.7	0.336	0.687 (0.319-1.477)
	AA	41	33.3	34	30.1	0.262	1.398 (0.779-2.510)
**rs2071219 dominant**	GG	28	22.8	16	14.2	Ref.	1.000
	AG+AA	195	77.2	97	85.8	0.052	0.491 (0.240-1.006)
**rs2071219 recessive**	AA	41	33.3	34	30.1	Ref.	1.000
	AG+GG	82	66.7	79	69.9	0.262	0.715 (0.398-1.284)

Table S44. Distribution of ALK1 rs2071219 genotypes according to the caliber of left retinal veins.

SNP	Genotype	< average		> average		P value	OR (95% CI)
		N	%	N	%		
**rs2071219**	GG	21	18.3	25	21.9	Ref.	1.000
	AG	61	53.0	46	40.4	0.843	1.081 (0.500-2.340)
	AA	33	28.7	43	37.7	0.278	0.707 (0.378-1.323)
**rs2071219 dominant**	GG	21	18.3	25	21.9	Ref.	1.000
	AG+AA	94	81.7	89	78.1	0.246	1.529 (0.746-3.136)
**rs2071219 recessive**	AA	33	28.7	43	37.7	Ref.	1.000
	AG+GG	82	71.3	71	62.3	0.278	1.414 (0.756-2.646)

Table S45. Distribution of ALK1 rs2071219 genotypes according to the caliber of right retinal veins.

SNP	Genotype	< average		> average		P value	OR (95% CI)
		N	%	N	%		
**rs2071219**	GG	21	18.6	19	19.2	Ref.	1.000
	AG	60	53.1	42	42.4	0.483	0.757 (0.347-1.651)
	AA	32	28.3	38	38.4	0.111	0.605 (0.326-1.123)
**rs2071219 dominant**	GG	21	18.6	19	19.2	Ref.	1.000
	AG+AA	92	81.4	80	80.8	0.554	1.251 (0.596-2.627)
**rs2071219 recessive**	AA	32	28.3	38	38.4	Ref.	1.000
	AG+GG	81	71.7	61	61.6	0.111	1.653 (0.890-3.070)

Table S46. Distribution of ALK1 rs2071219 genotypes according to the caliber of medium retinal veins.

SNP	Genotype	< average		> average		P value	OR (95% CI)
		N	%	N	%		
**rs2071219**	GG	23	17.3	22	21.0	Ref.	1.000
	AG	73	54.9	44	41.9	0.795	0.907 (0.433-1.898)
	AA	37	27.8	39	37.1	0.069	0.580 (0.323-1.044)
**rs2071219 dominant**	GG	23	17.3	22	21.0	Ref.	1.000
	AG+AA	110	82.7	83	79.0	0.209	1.563 (0.779-3.135)
**rs2071219 recessive**	AA	37	27.8	39	37.1	Ref.	1.000
	AG+GG	96	72.2	66	62.9	0.069	1.723 (0.958-3.099)

Table S47. Distribution of ALK1 rs2071219 genotypes according to the caliber of major retinal veins.

SNP	Genotype	< average		> average		P value	OR (95% CI)
		N	%	N	%		
**rs2071219**	GG	20	16.1	25	21.9	Ref.	1.000
	AG	71	57.3	46	40.4	0.917	0.961 (0.457-2.024)
	AA	33	26.6	43	37.7	0.017	0.486 (0.269-0.877)
**rs2071219 dominant**	GG	20	16.1	25	21.9	Ref.	1.000
	AG+AA	104	83.9	89	78.1	0.056	1.978 (0.983-3.979)
**rs2071219 recessive**	AA	33	26.6	43	37.7	Ref.	1.000
	AG+GG	91	73.4	71	62.3	0.017	2.057 (1.141-3.711)

KIDNEY DISEASE

Table S48. Distribution of ALK1 rs2071219 genotypes according to the presence of pathological albumin/creatinine index

SNP	Genotype	Normal		Pathological		P value	OR (95% CI)
		N	%	N	%		
**rs2071219**	GG	66	19.1	6	25.0	Ref.	1.000
	AG	182	52.4	9	37.5	0.957	0.970 (0.327-2.883)
	AA	99	28.5	9	37.5	0.192	0.527 (0.201-1.380)
**rs2071219 dominant**	GG	66	19.0	6	25.0	Ref.	1.000
	AG+AA	281	81.0	18	75.0	0.180	1.561 (0.814-2.994)
**rs2071219 recessive**	AA	99	28.5	9	37.5	Ref.	1.000
	AG+GG	248	71.5	15	62.5	0.062	1.692 (0.974-2.938)

Table S49. Distribution of ALK1 rs2071219 genotypes according to the presence of pathological kidney disease (evaluated by CKD-EPI)

SNP	Genotype	Normal		Pathological		P value	OR (95% CI)
		N	%	N	%		
**rs2071219**	GG	35	20.5	38	18.3	Ref.	1.000
	AG	91	53.2	104	50.0	0.346	0.735 (0.388-1.394)
	AA	45	26.3	66	31.7	0.178	0.706 (0.424-1.173)
**rs2071219 dominant**	GG	35	20.5	38	18.3	Ref.	1.000
	AG+AA	136	79.5	170	81.7	0.267	1.842 (0.626-5.421)
**rs2071219 recessive**	AA	45	26.3	66	31.7	Ref.	1.000
	AG+GG	126	73.7	142	68.3	0.192	1.898 (0.725-4.974)

HEART RATE

Table S50. Distribution of ALK1 rs2071219 genotypes according to the values of heart rate (cutpoint: 100 bpm)

SNP	Genotype	< 100		> 100		P value	OR (95% CI)
		N	%	N	%		
**rs2071219**	GG	72	19.1	1	33.3	Ref.	1.000
	AG	194	51.6	1	33.3	0.777	1.496 (0.092-24.360)
	AA	110	29.3	1	33.3	0.690	0.568 (0.035-9.192)
**rs2071219 dominant**	GG	72	19.1	1	33.3	Ref.	1.000
	AG+AA	304	80.9	2	66.7	0496	2.635 (0.162-42.875)
**rs2071219 recessive**	AA	110	29.3	1	33.3	Ref.	1.000
	AG+GG	266	70.7	2	66.7	0.690	1.761 (0.109-28.502)

Table S51. Distribution of ALK1 rs2071219 genotypes according to the values of heart rate (cutpoint: 90 bpm)

SNP	Genotype	< 90		> 90		P value	OR (95% CI)
		N	%	N	%		
**rs2071219**	GG	71	19.2	2	20.0	Ref.	1.000
	AG	191	51.8	4	40.0	0.754	0.759 (0.135-4.261)
	AA	107	29.0	4	40.0	0.417	0.559 (0.137-2.280)
**rs2071219 dominant**	GG	71	19.2	2	20.0	Ref.	1.000
	AG+AA	298	80.8	8	80.0	0.727	1.359 (0.243-7.601)
**rs2071219 recessive**	AA	107	29.0	4	40.0	Ref.	1.000
	AG+GG	262	71.0	6	60.0	0.417	1.790 (0.439-7.308)

Table S52. Distribution of ALK1 rs2071219 genotypes according to the values of heart rate (cutpoint: 70 bpm)

SNP	Genotype	< 70		> 70		P value	OR (95% CI)
		N	%	N	%		
**rs2071219**	GG	37	17.3	36	21.8	Ref.	1.000
	AG	115	53.7	80	48.5	0.483	1.237 (0.682-2.244)
	AA	62	29.0	49	29.7	0.620	0.887 (0.553-2.423)
**rs2071219 dominant**	GG	37	17.3	36	21.8	Ref.	1.000
	AG+AA	177	82.7	129	78.2	0.230	1.395 (0.810-2.401)
**rs2071219 recessive**	AA	62	29.0	49	29.7	Ref.	1.000
	AG+GG	152	71.0	116	70.3	0.620	1.127 (0.703-1.809)

Table S53. Distribution of ALK1 rs2071219 genotypes according to the values of heart rate (cutpoint: 50 bpm)

SNP	Genotype	< 50		> 50		P value	OR (95% CI)
		N	%	N	%		
**rs2071219**	GG	-	-	73	19.5	Ref.	1.000
	AG	1	25.0	194	51.7	0.997	43476623 (0.000-)
	AA	3	75.0	108	28.8	0.131	5.858 (0.591-58.111)
**rs2071219 dominant**	GG	-	-	73	19.5	Ref.	1.000
	AG+AA	4	100.0	302	80.5	0.997	19628223 (0.000-)
**rs2071219 recessive**	AA	3	75.0	108	28.8	Ref.	1.000
	AG+GG	1	25.0	267	71.2	0.131	0.171 (0.017-1.693)

2. ALK1 rs3847859 polymorphism

MEDICAL RECORD

Table S54. Distribution of ALK1 rs3847859 genotypes according to the presence of previous history of cardiovascular disease

SNP	Genotype	No		Yes		P value	OR (95% CI)
		N	%	N	%		
**rs3847859**	GG	114	31.5	6	35.3	Ref.	1.000
	AG	197	54.4	6	35.3	0.348	0.552 (0.160-1.910)
	AA	51	14.1	5	29.4	0.058	0.303 (0.088-1.041)
**rs3847859 dominant**	GG	114	31.5	6	35.3	Ref.	1.000
	AG+AA	248	68.5	11	64.7	0.313	1.820 (0.569-5.830)
**rs3847859 recessive**	AA	51	14.1	5	29.4	Ref.	1.000
	AG+GG	311	85.9	12	70.6	0.058	3.296 (0.960-11.311)

Table S55. Distribution of ALK1 rs3847859 genotypes according to the presence of previous history of premature cardiovascular disease

SNP	Genotype	No		Yes		P value	OR (95% CI)
		N	%	N	%		
**rs3847859**	GG	114	31.5	6	35.3	Ref.	1.000
	AG	197	54.4	6	35.3	0.348	0.552 (0.160-1.910)
	AA	51	14.1	5	29.4	0.058	0.303 (0.088-1.041)
**rs3847859 dominant**	GG	114	31.5	6	35.3	Ref.	1.000
	AG+AA	248	68.5	11	64.7	0.313	1.820 (0.569-5.820)
**rs3847859 recessive**	AA	51	14.1	5	29.4	Ref.	1.000
	AG+GG	311	85.9	11	64.7	0.058	3.296 (0.960-11.311)

Table S56. Distribution of ALK1 rs3847859 genotypes according to the presence of previous history of cerebrovascular disease

SNP	Genotype	No		Yes		P value	OR (95% CI)
		N	%	N	%		
**rs3847859**	GG	119	31.6	1	33.3	Ref.	1.000
	AG	201	53.5	2	66.7	0.997	9959091.47 (0.000-)
	AA	56	14.9	-	-	-	-
**rs3847859 dominant**	GG	119	31.6	1	33.3	Ref.	1.000
	AG+AA	257	68.4	2	66.7	0.800	0.730 (0.064-8.278)
**rs3847859 recessive**	AA	56	14.9	-	-	Ref.	1.000
	AG+GG	320	85.1	3	100.0	0.997	0.000 (0.000-)

Table S57. Distribution of ALK1 rs3847859 genotypes according to the presence of previous history of heart failure

SNP	Genotype	No		Yes		P value	OR (95% CI)
		N	%	N	%		
**rs3847859**	GG	115	31.2	5	50.0	Ref.	1.000
	AG	200	54.2	3	30.0	0.843	1.189 (0.216-6.533)
	AA	54	14.6	2	20.0	0.341	0.409 (0.065-0.573)
**rs3847859 dominant**	GG	115	31.2	5	50.0	Ref.	1.000
	AG+AA	254	68.8	5	50.0	0.156	2.905 (0.667-12.657)
**rs3847859 recessive**	AA	54	14.6	2	20.0	Ref.	1.000
	AG+GG	315	85.4	8	80.0	0.341	2.444 (0.389-15.364)

Table S58. Distribution of ALK1 rs3847859 genotypes according to the presence of previous personal history of dislipemia.

SNP	Genotype	No		Yes		P value	OR (95% CI)
		N	%	N	%		
**rs3847859**	GG	41	34.2	79	30.5	Ref.	1.000
	AG	60	50.0	143	55.2	0.980	0.991 (0.500-1.966)
	AA	19	15.8	37	14.3	0.584	1.197 (0.629-2.275)
**rs3847859 dominant**	GG	41	34.2	79	30.5	Ref.	1.000
	AG+AA	79	65.8	180	69.5	0.455	0.828 (0.506-1.357)
**rs3847859 recessive**	AA	19	15.8	37	14.3	Ref.	1.000
	AG+GG	101	84.2	222	85.7	0.584	0.836 (0.439-1.589)

Table S59. Distribution of ALK1 rs3847859 genotypes according to the presence of previous personal history of diabetes.

SNP	Genotype	No		Yes		P value	OR (95% CI)
		N	%	N	%		
**rs3847859**	GG	88	30.2	32	36.4	Ref.	1.000
	AG	163	56.0	40	45.5	0.713	0.873 (0424-1.797)
	AA	40	13.7	16	18.2	0.149	0.603 (0.303-1.200)
**rs3847859 dominant**	GG	88	30.2	32	36.4	Ref.	1.000
	AG+AA	203	69.8	56	63.6	0.181	1.448 (0.842-2490)
**rs3847859 recessive**	AA	40	13.7	16	18.2	Ref.	1.000
	AG+GG	251	86.3	72	81.8	0.149	1.658 (0.834-3.298)

Table S60. Distribution of ALK1 rs3847859 genotypes according to the presence of previous personal history of hypertension.

SNP	Genotype	No		Yes		P value	OR (95% CI)
		N	%	N	%		
**rs3847859**	GG	32	31.4	88	31.8	Ref.	1.000
	AG	50	49.0	153	55.2	0.232	1.534 (0.761-3.093)
	AA	20	19.6	36	13.0	0.112	1.697 (0.885-3.254)
**rs3847859 dominant**	GG	32	31.4	88	31.8	Ref.	1.000
	AG+AA	70	68.6	189	68.2	0.709	0.904 (0.532-1.536)
**rs3847859 recessive**	AA	20	19.6	36	13.0	Ref.	1.000
	AG+GG	82	80.4	241	87.0	0.112	0.589 (0.307-1.130)

Table S61. Distribution of ALK1 rs3847859 genotypes according to the presence of total peripheral arterial disease

SNP	Genotype	No		Yes		P value	OR (95% CI)
		N	%	N	%		
**rs3847859**	GG	112	32.2	1	20.0	Ref.	1.000
	AG	185	53.1	3	60.0	0.481	0.362 (0.021-6.127)
	AA	51	14.7	1	20.0	0.815	0.758 (0.074-7.775)
**rs3847859 dominant**	GG	112	32.2	1	20.0	Ref.	1.000
	AG+AA	236	67.8	4	80.0	0.528	0.477 (0.048-4.738)
**rs3847859 recessive**	AA	51	14.7	1	20.0	Ref.	1.000
	AG+GG	297	85.3	4	80.0	0.815	1.319 (0.129-13.536)

Table S62. Distribution of ALK1 rs3847859 genotypes according to the presence of left ventricular hypertrophy (evaluated by electrocardiography)

SNP	Genotype	No		Yes		P value	OR (95% CI)
		N	%	N	%		
**rs3847859**	GG	107	31.3	10	33.3	Ref.	1.000

	AG	182	53.2	17	56.7	0.469	1.636 (0.431-6.208)
	AA	53	15.5	3	10.0	0.431	1.663 (0.469-5.896)
**rs3847859 dominant**	GG	107	31.3	10	33.3	Ref.	1.000
	AG+AA	235	68.7	20	66.7	0.970	0.984 (0.433-2.235)
**rs3847859 recessive**	AA	53	15.5	3	10.0	Ref.	1.000
	AG+GG	289	84.5	27	90.0	0.431	0.601 (0.170-2.133)

Table S63. Distribution of ALK1 rs3847859 genotypes according to the presence of left ventricular hypertrophy (evaluated by Cornell voltage duration product)

SNP	Genotype	No		Yes		P value	OR (95% CI)
		N	%	N	%		
**rs3847859**	GG	113	32.4	4	17.4	Ref.	1.000
	AG	184	52.7	16	69.6	0.619	0.681 (0.143-3.235)
	AA	52	14.9	3	13.0	0.553	1.480 (0.405-5.409)
**rs3847859 dominant**	GG	113	32.4	4	17.4	Ref.	1.000
	AG+AA	236	67.6	19	82.6	0.181	0.460 (0.147-1.437)
**rs3847859 recessive**	AA	52	14.9	3	13.0	Ref.	1.000
	AG+GG	297	85.1	20	87.0	0.553	0.676 (0.185-2.469)

Table S64. Distribution of ALK1 rs3847859 genotypes according to the presence of left ventricular hypertrophy (evaluated by Sokolow-Lyon index)

SNP	Genotype	No		Yes		P value	OR (95% CI)
		N	%	N	%		
**rs3847859**	GG	119	31.9	1	20.0	Ref.	1.000
	AG	198	53.1	4	80.0	0.998	1.201 (0.000-)
	AA	56	15.0	-	-	-	-
**rs3847859 dominant**	GG	119	31.9	1	20.0	Ref.	1.000
	AG+AA	254	68.1	4	80.0	0.380	0.371 (0.040-3.397)
**rs3847859 recessive**	AA	56	15.0	-	-	Ref.	1.000
	AG+GG	317	85.0	5	100.0	0.997	1.008 (0.000-)

Table S65. Distribution of ALK1 rs2071219 genotypes according to the presence of left ventricular hypertrophy (evaluated by Lewis index)

SNP	Genotype	No		Yes		P value	OR (95% CI)
		N	%	N	%		
**rs3847859**	GG	66	28.6	6	17.6	Ref.	1.000
	AG	134	58.0	24	70.6	0.519	0.641 (0.166-2.473)
	AA	31	13.4	4	11.8	0.546	1.422 (0.454-4.456)
**rs3847859 dominant**	GG	66	28.6	6	17.6	Ref.	1.000
	AG+AA	165	71.4	28	82.4	0.102	0.451 (0.174-1.172)
**rs3847859 recessive**	AA	31	13.4	4	11.8	Ref.	1.000
	AG+GG	200	86.6	30	88.2	0.546	0.703 (0.224-2.204)

Table S66. Distribution of ALK1 rs3847859 genotypes according to the presence of abdominal obesity

SNP	Genotype	No		Yes		P value	OR (95% CI)
		N	%	N	%		
**rs3847859**	GG	55	31.3	65	32.0	Ref.	1.000
	AG	97	55.1	106	52.2	0.823	0.928 (0.484-1.780)
	AA	24	13.6	32	15.8	0.473	0.801 (0.436-1.470)
**rs3847859 dominant**	GG	55	31.3	65	32.0	Ref.	1.000
	AG+AA	121	68.7	138	68.0	0.531	1.159 (0.730-1.840)
	AA	24	13.6	32	15.8	Ref.	1.000

**rs3847859 recessive**	AG+GG	152	86.4	171	84.2	0.473	1.249 (0.680-2.292)

Table S67. Distribution of ALK1 rs3847859 genotypes according to the presence of metabolic syndrome

SNP	Genotype	No		Yes		P value	OR (95% CI)
		N	%	N	%		
**rs3847859**	GG	85	29.8	35	37.3	Ref.	1.000
	AG	160	56.2	43	45.8	0.897	0.953 (0.461-1.970)
	AA	40	14.0	16	17.9	0.266	0.675 (0.337-1.349)
**rs3847859 dominant**	GG	85	29.8	35	37.2	Ref.	1.000
	AG+AA	200	70.2	59	62.8	0.205	1.413 (0.828-2.412)
**rs3847859 recessive**	AA	40	14.0	16	17.0	Ref.	1.000
	AG+GG	245	86.0	78	83.0	0.266	1.482 (0.741-2.964)

ANALYTICAL VALUES

Table S68. Distribution of ALK1 rs3847859 genotypes according to the presence of elevated cholesterol plasma levels

SNP	Genotype	No		Yes		P value	OR (95% CI)
		N	%	N	%		
**rs3847859**	GG	28	24.6	90	35.7	Ref.	1.000
	AG	66	57.9	127	50.4	0.081	1.866 (0.925-3.766)
	AA	20	17.5	35	13.9	0.749	1.108 (0.589-2.084)
**rs3847859 dominant**	GG	28	24.6	90	35.7	Ref.	1.000
	AG+AA	86	75.4	162	64.3	0.051	1.684 (0.997-2.844)
**rs3847859 recessive**	AA	20	17.5	35	13.9	Ref.	1.000
	AG+GG	94	82.5	217	86.1	0.749	0.902 (0.480-1.696)

Table S69. Distribution of ALK1 rs3847859 genotypes according to the presence of reduced cholesterol-high density lipoprotein plasma levels

SNP	Genotype	No		Yes		P value	OR (95% CI)
		N	%	N	%		
**rs3847859**	GG	93	34.1	25	26.9	Ref.	1.000
	AG	139	65.9	54	58.0	0.665	0.843 (0.389-1.827)
	AA	41	15.0	14	15.1	0.773	1.109 (0.548-2.246)
**rs3847859 dominant**	GG	93	34.1	25	26.9	Ref.	1.000
	AG+AA	180	65.9	68	73.1	0.336	0.760 (0.434-1.329)
**rs3847859 recessive**	AA	41	15.0	14	15.1	Ref.	1.000
	AG+GG	232	85.0	79	84.9	0.773	0.902 (0.445-1.826)

Table S70. Distribution of ALK1 rs3847859 genotypes according to the presence of elevated cholesterol-low density lipoprotein plasma levels

SNP	Genotype	No		Yes		P value	OR (95% CI)
		N	%	N	%		
**rs3847859**	GG	35	30.7	84	32.4	Ref.	1.000
	AG	59	51.8	140	54.1	0.344	1.397 (0.699-2.790)
	AA	20	17.5	35	13.5	0.297	1.407 (0.740-2.674)
**rs3847859 dominant**	GG	35	30.7	84	32.4	Ref.	1.000
	AG+AA	79	69.3	175	67.6	0.997	0.993 (0.597-1.651)
**rs3847859 recessive**	AA	20	17.5	35	13.5	Ref.	1.000
	AG+GG	94	82.5	224	86.5	0.297	0.711 (0.374-1.351)

Table S71. Distribution of ALK1 rs3847859 genotypes according to the presence of elevated triglycerides plasma levels

SNP	Genotype	No		Yes		P value	OR (95% CI)
		N	%	N	%		
**rs3847859**	GG	93	32.9	27	28.1	Ref.	1.000
	AG	147	51.9	56	58.4	0.827	0.918 (0.428-1.973)
	AA	43	15.2	13	13.5	0.462	1.302 (0.645-2.627)
**rs3847859 dominant**	GG	93	32.9	27	28.1	Ref.	1.000
	AG+AA	190	67.1	69	71.9	0.203	0.706 (0.412-1.207)
**rs3847859 recessive**	AA	43	15.2	13	13.5	Ref.	1.000
	AG+GG	240	844.8	83	86.5	0.462	0.768 (0.381-1.551)

Table S72. Distribution of ALK1 rs3847859 genotypes according to the presence of dyslipemia

SNP	Genotype	No		Yes		P value	OR (95% CI)
		N	%	N	%		
**rs3847859**	GG	7	31.8	113	31.7	Ref.	1.000
	AG	11	50.0	191	53.7	0.692	1.302 (0.352-4.816)
	AA	4	18.2	52	14.6	0.656	1.319 (0.390-4.458)
**rs3847859 dominant**	GG	7	31.8	113	31.7	Ref.	1.000
	AG+AA	15	68.2	243	68.3	0.980	0.987 (0.363-2.684)
**rs3847859 recessive**	AA	4	18.2	52	14.6	Ref.	1.000
	AG+GG	18	81.8	304	85.4	0.656	0.758 (0.224-2.684)

Table S73. Distribution of ALK1 rs3847859 genotypes according to the presence of altered basal glycemia

SNP	Genotype	No		Yes		P value	OR (95% CI)
		N	%	N	%		
**rs3847859**	GG	104	31.1	16	35.6	Ref.	1.000
	AG	184	55.1	19	42.2	0.458	0.720 (0.303-1.712)
	AA	46	13.8	10	22.2	0.074	0.468 (0.203-1.077)
**rs3847859 dominant**	GG	104	31.1	16	35.6	Ref.	1.000
	AG+AA	230	68.9	29	64.4	0.235	1.538 (0.755-3.132)
**rs3847859 recessive**	AA	46	13.8	10	22.2	Ref.	1.000
	AG+GG	288	86.2	35	77.8	0.074	2.136 (0.928-4.917)

Table S74. Distribution of ALK1 rs3847859 genotypes according to the presence of microalbuminuria

SNP	Genotype	No		Yes		P value	OR (95% CI)
		N	%	N	%		
**rs3847859**	GG	111	31.6	7	35.0	Ref.	1.000
	AG	193	55.0	6	30.0	0.116	0.412 (0.136-1.247)
	AA	47	13.4	7	35.0	0.007	0.208 (0.067-0.651)
**rs3847859 dominant**	GG	111	31.6	7	35.0	Ref.	1.000
	AG+AA	240	68.4	13	65.0	0.233	1.977 (0.645-6.055)
**rs3847859 recessive**	AA	47	13.4	7	35.0	Ref.	1.000
	AG+GG	304	86.6	13	65.0	0.007	4.803 (1.536-15.017)

CARDIOVASCULAR TESTS

Table S75. Distribution of ALK1 rs3847859 genotypes according to the presence of elevated pulse pressure

SNP	Genotype	No		Yes		P value	OR (95% CI)
		N	%	N	%		
**rs3847859**	GG	91	31.7	29	31.5	Ref.	1.000
	AG	154	53.7	49	53.3	0.925	0.963 (0.443-2.094)
	AA	42	14.6	14	15.2	0.893	0.952 (0.462-1.959)
**rs3847859 dominant**	GG	91	31.7	29	31.5	Ref.	1.000
	AG+AA	196	68.3	63	68.5	0.966	1.012 (0.581-1.764)
**rs3847859 recessive**	AA	42	14.6	14	15.2	Ref.	1.000
	AG+GG	245	85.4	78	84.8	0.893	1.051 (0.510-2.163)

Table S76. Distribution of ALK1 rs3847859 genotypes according to the presence of increased carotid intima-media thickness

SNP	Genotype	No		Yes		P value	OR (95% CI)
		N	%	N	%		
**rs3847859**	GG	100	32.3	19	28.4	Ref.	1.000
	AG	165	53.2	37	55.2	0.476	0.736 (0.316-1.712)
	AA	45	14.5	11	16.4	0.830	0.919 (0.425-1.986)
**rs3847859 dominant**	GG	100	32.3	19	28.4	Ref.	1.000
	AG+AA	210	67.7	48	71.6	0.483	0.800 (0.429-1.492)
**rs3847859 recessive**	AA	45	14.5	11	16.4	Ref.	1.000
	AG+GG	265	85.5	56	83.6	0.830	1.088 (0.504-2.350)

Table S77. Distribution of ALK1 rs2071219 genotypes according to the values of pulse wave velocity

SNP	Genotype	< average		➢ average		P value	OR (95% CI)
		N	%	N	%		
**rs3847859**	GG	102	32.2	17	29.8	Ref.	1.000
	AG	168	53.0	32	56.1	0.718	1.224 (0.408-3.671)
	AA	47	14.8	8	14.0	0.565	1.348 (0.488-3.726)
**rs3847859 dominant**	GG	102	32.2	17	29.8	Ref.	1.000
	AG+AA	215	67.8	40	70.2	0.807	0.908 (0.419-1.967)
**rs3847859 recessive**	AA	47	14.8	8	14.0	Ref.	1.000
	AG+GG	270	67.8	49	86.0	0.565	0.742 (0.268-2.050)

Table S78. Distribution of ALK1 rs3847859 genotypes according to the presence of pathological ankle-brachial index

SNP	Genotype	No		Yes		P value	OR (95% CI)
		N	%	N	%		
**rs3847859**	GG	119	31.9	1	20.0	Ref.	1.000
	AG	199	53.4	3	60.0	0.541	0.415 (0.025-6.979)
	AA	55	14.7	1	20.0	0.903	0.865 (0.085-8.814)
**rs3847859 dominant**	GG	119	31.9	1	20.0	Ref.	1.000
	AG+AA	254	68.1	4	80.0	0.530	0.479 (0.048-4.753)
**rs3847859 recessive**	AA	55	14.7	1	20.0	Ref.	1.000
	AG+GG	318	85.3	4	80.0	0.903	1.156 (0.113-11.771)

CURRENT DISEASES

Table S79. Distribution of ALK1 rs3847859 genotypes according to the presence of cerebrovascular disease

SNP	Genotype	No		Yes		P value	OR (95% CI)
		N	%	N	%		
**rs3847859**	GG	119	31.6	1	33.3	Ref.	1.000
	AG	201	53.5	2	66.7	0.997	9959091.47 (0.000-)
	AA	56	14.9	-	-	-	-
**rs3847859 dominant**	GG	119	31.6	1	33.3	Ref.	1.000
	AG+AA	257	68.4	2	66.7	0.800	0.730 (0.064-8.270)
**rs3847859 recessive**	AA	56	14.9	-	-	Ref.	1.000
	AG+GG	320	85.1	3	100.0	0.997	0.000 (0.000-)

Table S80. Distribution of ALK1 rs3847859 genotypes according to the presence of cardiovascular disease

SNP	Genotype	No		Yes		P value	OR (95% CI)
		N	%	N	%		
**rs3847859**	GG	115	31.2	5	50.0	Ref.	1.000
	AG	200	54.2	3	30.0	0.843	1.189 (0.216-6.533)
	AA	54	14.6	2	20.0	0.341	0.409 (0.065-2.573)
**rs3847859 dominant**	GG	115	31.2	5	50.0	Ref.	1.000
	AG+AA	254	68.8	5	50.0	0.156	2.905 (0.667-12.657)
**rs3847859 recessive**	AA	54	14.6	2	20.0	Ref.	1.000
	AG+GG	315	85.4	8	80.0	0.341	2.444 (0.389-15.364)

Table S81. Distribution of ALK1 rs3847859 genotypes according to the presence of kidney disease

SNP	Genotype	No		Yes		P value	OR (95% CI)
		N	%	N	%		
**rs3847859**	GG	115	31.3	3	75.0	Ref.	1.000
	AG	198	54.0	1	25.0	0.997	32827753.3 (0.000-)
	AA	54	14.7	-	-	-	-
**rs3847859 dominant**	GG	115	31.3	3	75.0	Ref.	1.000
	AG+AA	252	68.7	1	25.0	0.189	4.652 (0.469-46.105)
**rs3847859 recessive**	AA	54	14.7	-	-	Ref.	1.000
	AG+GG	313	85.3	4	100.0	0.997	0.000 (0.000-)

Table S82. Distribution of ALK1 rs3847859 genotypes according to the presence of advanced retinopathy

SNP	Genotype	No		Yes		P value	OR (95% CI)
		N	%	N	%		
**rs3847859**	GG	44	34.1	2	25.0	Ref.	1.000
	AG	64	49.6	5	62.5	0.978	0.965 (0.079-11.751)
	AA	21	16.3	1	12.5	0.599	1.829 (0.193-17.325)
**rs3847859 dominant**	GG	44	34.1	2	25.0	Ref.	1.000
	AG+AA	85	65.9	6	75.0	0.461	0.528 (0.096-2.889)
**rs3847859 recessive**	AA	21	16.3	1	12.5	Ref.	1.000
	AG+GG	108	83.7	7	87.5	0.599	0.547 (0.058-5.178)

Table S83. Distribution of ALK1 rs3847859 genotypes according to the presence of controlled arterial pressure.

SNP	Genotype	No		Yes		P value	OR (95% CI)
		N	%	N	%		
**rs3847859**	GG	19	34.5	17	41.5	Ref.	1.000
	AG	28	50.9	19	46.3	0.583	1.447 (0.387-5.414)

	AA	8	14.	5	12.2	0.881	1.105 (0.302-4.046)
**rs3847859 dominant**	GG	19	34.5	17	41.5	Ref.	1.000
	AG+AA	36	65.5	24	58.5	0.555	1.310 (0.534-3.214)
**rs3847859 recessive**	AA	8	14.5	5	12.2	Ref.	1.000
	AG+GG	47	85.5	36	87.8	0.881	0.905 (0.247-3.315)

Table S84. Distribution of ALK1 rs3847859 genotypes according to the presence of target organ damage.

SNP	Genotype	No		Yes		P value	OR (95% CI)
		N	%	N	%		
**rs3847859**	GG	23	38.3	13	38.2	Ref.	1.000
	AG	28	46.7	17	50.0	0.733	1.269 (0.323-4.978)
	AA	9	15.0	4	11.8	0.649	1.366 (0.357-5.233)
**rs3847859 dominant**	GG	23	38.3	13	38.2	Ref.	1.000
	AG+AA	37	61.7	21	61.8	0.875	0.929 (0.371-2.328)
**rs3847859 recessive**	AA	9	15.0	4	11.8	Ref.	1.000
	AG+GG	51	85.0	30	88.2	0.649	0.732 (0.191-2.805)

Table S85. Distribution of ALK1 rs3847859 genotypes according to the presence of hypertension, diabetes and obesity

SNP	Genotype	No		Yes		P value	OR (95% CI)
		N	%	N	%		
**rs3847859**	GG	34	28.1	86	33.3	Ref.	1.000
	AG	67	55.4	136	52.7	0.362	1.374 (0.694-2.717)
	AA	20	16.5	36	14.0	0.708	1.127 (0.603-2.108)
**rs3847859 dominant**	GG	34	28.1	86	33.3	Ref.	1.000
	AG+AA	87	71.9	172	66.7	0.437	1.219 (0.740-2.007)
**rs3847859 recessive**	AA	20	16.5	36	14.0	Ref.	1.000
	AG+GG	101	83.5	222	86.0	0.708	0.887 (0.474-1.659)

Table S86. Distribution of ALK1 rs3847859 genotypes according to the presence of hypertension and diabetes

SNP	Genotype	No		Yes		P value	OR (95% CI)
		N	%	N	%		
**rs3847859**	GG	48	30.4	72	32.6	Ref.	1.000
	AG	84	53.1	119	53.8	0.483	1.262 (0.658-2.422)
	AA	26	16.5	30	13.6	0.497	1.233 (0.673-2.260)
**rs3847859 dominant**	GG	48	30.4	72	32.6	Ref.	1.000
	AG+AA	110	69.6	149	67.4	0.922	1.024 (0.640-1.636)
**rs3847859 recessive**	AA	26	16.5	30	13.6	Ref.	1.000
	AG+GG	132	83.5	191	86.4	0.497	0.811 (0.442-1.486)

RISK SCALES

Table S87. Distribution of ALK1 rs3847859 genotypes according to the presence of cardiovascular risk

SNP	Genotype	Low		High		P value	OR (95% CI)
		N	%	N	%		
**rs3847859**	GG	10	32.3	108	32.2	Ref.	1.000
	AG	15	48.3	178	53.2	0.659	1.328 (0.376-4.696)
	AA	6	19.4	49	14.6	0.725	1.232 (0.385-3.940)
**rs3847859 dominant**	GG	10	32.3	108	32.2	Ref.	1.000
	AG+AA	21	67.7	227	67.8	0.878	1.078 (0.415-2.803)

**rs3847859 recessive**	AA	6	19.4	49	14.6	Ref.	1.000
	AG+GG	25	80.6	286	85.4	0.725	0.811 (0.254-2.594)

Table S88. Distribution of ALK1 rs3847859 genotypes according to the presence of diabetes (based on 2013 guidelines)

SNP	Genotype	No		Yes		P value	OR (95% CI)
		N	%	N	%		
**rs3847859**	GG	95	30.1	25	39.7	Ref.	1.000
	AG	176	55.7	27	42.9	0.928	1.038 (0.463-2.327)
	AA	45	14.2	11	17.5	0.234	0.620 (0.283-1.362)
**rs3847859 dominant**	GG	95	30.1	25	39.7	Ref.	1.000
	AG+AA	221	69.9	38	60.3	0.097	1.673 (0.911-3.075)
**rs3847859 recessive**	AA	45	14.2	11	17.5	Ref.	1.000
	AG+GG	271	85.8	52	82.5	0.234	1.612 (0.734.3.538)

Table S89. Distribution of ALK1 rs2071219 genotypes according to the presence of hypertension (based on 2013 guidelines)

SNP	Genotype	No		Yes		P value	OR (95% CI)
		N	%	N	%		
**rs3847859**	GG	59	30.3	61	33.2	Ref.	1.000
	AG	105	53.8	98	53.2	0.486	1.257 (0.660-2.393)
	AA	31	15.9	25	13.6	0.635	1.156 (0.634-2.108)
**rs3847859 dominant**	GG	59	30.3	61	33.2	Ref.	1.000
	AG+AA	136	69.7	123	66.8	0.721	1.087 (0.688-1.717)
**rs3847859 recessive**	AA	31	15.9	25	13.6	Ref.	1.000
	AG+GG	164	84.1	159	86.4	0.635	0.865 (0.474-1.576)

RETINOPATHY

Table S90. Distribution of ALK1 rs2071219 genotypes according to the caliber of medium retinal arteries

SNP	Genotype	< average		> average		P value	OR (95% CI)
		N	%	N	%		
**rs3847859**	GG	44	35.2	44	38.9	Ref.	1.000
	AG	65	52.0	54	47.8	0.874	1.069 (0.470-2.430)
	AA	16	12.8	15	13.3	0.772	0.889 (0.402-1.966)
**rs3847859 dominant**	GG	44	35.2	44	38.9	Ref.	1.000
	AG+AA	81	64.8	69	61.1	0.515	1.202 (0.691-2.091)
**rs3847859 recessive**	AA	16	12.8	15	13.3	Ref.	1.000
	AG+GG	109	87.2	98	86.7	0.772	1.124 (0.509-2.485)

Table S91. Distribution of ALK1 rs3847859 genotypes according to the caliber of right retinal arteries

SNP	Genotype	< average		> average		P value	OR (95% CI)
		N	%	N	%		
**rs3847859**	GG	39	36.1	39	37.5	Ref.	1.000
	AG	56	51.9	52	50.0	0.995	0.997 (0.409-2.433)
	AA	13	12.0	13	12.5	0.870	0.931 (0.394-2.198)
**rs3847859 dominant**	GG	39	36.1	39	37,5	Ref.	1.000
	AG+AA	69	63.9	65	62.5	0.818	1.071 (0.597-1.924)
**rs3847859 recessive**	AA	13	12.0	13	12.5	Ref.	1.000
	AG+GG	95	88.0	91	87.5	0.870	1.074 (0.455-2.538)

Table S92. Distribution of ALK1 rs2071219 genotypes according to the caliber of minor retinal arteries

SNP	Genotype	< average		> average		P value	OR (95% CI)
		N	%	N	%		
**rs3847859**	GG	43	35.0	45	39.1	Ref.	1.000
	AG	64	52.0	55	47.8	0.836	1.091 (0.479-2.486)
	AA	16	13.0	15	13.0	0.825	0.914 (0.413-2.025)
**rs3847859 dominant**	GG	43	35.0	45	39.1	Ref.	1.000
	AG+AA	80	65.0	70	60.9	0.532	1.194 (0.685-2.079)
**rs3847859 recessive**	AA	16	13.0	15	13.0	Ref.	1.000
	AG+GG	107	87.0	100	87.0	0.825	1.094 (0.494-2.422)

Table S93. Distribution of ALK1 rs2071219 genotypes according to the caliber of left retinal arteries

SNP	Genotype	< average		> average		P value	OR (95% CI)
		N	%	N	%		
**rs3847859**	GG	38	36.5	42	39.3	Ref.	1.000
	AG	53	51.0	49	45.8	0.805	0.897 (0.378-2.128)
	AA	13	12.5	16	15.0	0.506	0.752 (0.325-1.740)
**rs3847859 dominant**	GG	38	36.5	42	39.3	Ref.	1.000
	AG+AA	66	63.5	65	60.7	0.563	1.192 (0.658-2.160)
**rs3847859 recessive**	AA	13	12.5	16	15.0	Ref.	1.000
	AG+GG	91	87.5	91	85.0	0.506	1.329 (0.575-3.073)

Table S94. Distribution of ALK1 rs3847859 genotypes according to the values of left arteriovenous index (AVIx)

SNP	Genotype	< average		> average		P value	OR (95% CI)
		N	%	N	%		
**rs3847859**	GG	30	35.7	40	43.5	Ref.	1.000
	AG	44	52.4	39	42.4	0.976	0.985 (0.376-2.584)
	AA	10	11.9	13	14.1	0.390	0.662 (0.238-1.696)
**rs3847859 dominant**	GG	30	35.7	40	43.5	Ref.	1.000
	AG+AA	54	64.3	52	56.5	0.229	1.488 (0.778-2.846)
**rs3847859 recessive**	AA	10	11.9	13	14.1	Ref.	1.000
	AG+GG	74	88.1	79	85.9	0.390	1.510 (0.590-3.868)

Table S95. Distribution of ALK1 rs3847859 genotypes according to the values of right arteriovenous index (AVIx)

SNP	Genotype	< average		> average		P value	OR (95% CI)
		N	%	N	%		
**rs3847859**	GG	29	35.4	34	43.0	Ref.	1.000
	AG	44	53.6	34	43.0	0.962	0.976 (0.353-2.697)
	AA	9	11.0	11	14.0	0.371	0.636 (0.236-1.714)
**rs3847859 dominant**	GG	29	35.4	34	43.0	Ref.	1.000
	AG+AA	53	64.6	45	57.0	0.210	1.535 (0.785-3.000)
**rs3847859 recessive**	AA	9	11.0	11	13.9	Ref.	1.000
	AG+GG	73	89.0	68	86.1	0.371	1.573 (0.583-4.243)

Table S96. Distribution of ALK1 rs3847859 genotypes according to the values of medium arteriovenous index (AVIx)

SNP	Genotype	< average		> average		P value	OR (95% CI)
		N	%	N	%		
**rs3847859**	GG	43	35.0	44	38.9	Ref.	1.000
	AG	64	52.0	54	47.8	0.820	1.100 (0.483-2.506)
	AA	16	13.0	15	13.3	0.801	0.903 (0.408-1.998)
**rs3847859 dominant**	GG	43	35.0	44	38.9	Ref.	1.000
	AG+AA	80	65.0	69	61.1	0.486	1.219 (0.698-2.128)
**rs3847859 recessive**	AA	16	13.0	15	13.3	Ref.	1.000
	AG+GG	107	87.0	98	86.7	0.801	1.108 (0.501-2.451)

Table S97. Distribution of ALK1 rs2071219 genotypes according to the caliber of left retinal veins

SNP	Genotype	< average		> average		P value	OR (95% CI)
		N	%	N	%		
**rs3847859**	GG	45	39.1	35	36.5	Ref.	1.000
	AG	56	48.7	46	47.9	0.468	0.729 (0.311-1.711)
	AA	14	12.2	15	15.6	0.523	0.764 (0.334-1.746)
**rs3847859 dominant**	GG	45	39.1	35	36.5	Ref.	1.000
	AG+AA	70	60.9	61	63.5	0.877	0.954 (0.528-1.724)
**rs3847859 recessive**	AA	14	12.2	15	15.6	Ref.	1.000
	AG+GG	101	87.8	81	84.4	0.523	1.309 (0.573-2.994)

Table S98. Distribution of ALK1 rs2071219 genotypes according to the caliber of right retinal veins

SNP	Genotype	< average		> average		P value	OR (95% CI)
		N	%	N	%		
**rs3847859**	GG	44	38.9	34	34.3	Ref.	1.000
	AG	54	47.8	54	54.5	0.924	1.045 (0.424-2.575)
	AA	15	13.3	11	11.1	0.481	1.366 (0.574-3.252)
**rs3847859 dominant**	GG	44	38.9	34	34.3	Ref.	1.000
	AG+AA	69	61.1	65	65.7	0.372	0.765 (0.425-1.377)
**rs3847859 recessive**	AA	15	13.3	11	11.1	Ref.	1.000
	AG+GG	98	86.7	88	88.9	0.481	0.732 (0.308-1.743)

Table S99. Distribution of ALK1 rs2071219 genotypes according to the caliber of medium retinal veins

SNP	Genotype	< average		> average		P value	OR (95% CI)
		N	%	N	%		
**rs3847859**	GG	50	37.6	38	36.2	Ref.	1.000
	AG	68	51.1	51	48.6	0.399	0.702 (0.308-1.598)
	AA	15	11.3	16	15.2	0.380	0.791 (0.317-1.550)
**rs3847859 dominant**	GG	50	37.6	38	36.2	Ref.	1.000
	AG+AA	83	62.4	67	63.8	0.997	1.001 (0.573-1.750)
**rs3847859 recessive**	AA	15	11.3	16	15.2	Ref.	1.000
	AG+GG	118	88.7	89	84.8	0.380	1.427 (0.645-3.156)

Table S100. Distribution of ALK1 rs2071219 genotypes according to the caliber of major retinal veins

SNP	Genotype	< average		> average		P value	OR (95% CI)
		N	%	N	%		
**rs3847859**	GG	47	37.9	41	36.0	Ref.	1.000
	AG	59	47.6	60	52.6	0.637	1.221 (0.533-2.798)

	AA	18	14.5	13	11.4	0.402	1.408 (0.632-3.133)
**rs3847859 dominant**	GG	47	37.9	41	36.0	Ref.	1.000
	AG+AA	77	62.1	73	64.0	0.614	0.867 (0.499-1.508)
**rs3847859 recessive**	AA	18	14.5	13	11.4	Ref.	1.000
	AG+GG	106	85.5	101	88.6	0.402	0.710 (0.319-1.581)

KIDNEY DISEASE

Table S101. Distribution of ALK1 rs3847859 genotypes according to the presence of pathological albumin/creatinine index

SNP	Genotype	Normal		Pathological		P value	OR (95% CI)
		N	%	N	%		
**rs3847859**	GG	108	31.1	10	41.7	Ref.	1.000
	AG	192	55.4	7	29.1	0.329	0.597 (0.211-1.684)
	AA	47	13.5	7	29.2	0.012	0.243 (0.081-0.733)
**rs3847859 dominant**	GG	108	31.1	10	41.7	Ref.	1.000
	AG+AA	239	68.9	14	58.3	0.079	2.455 (0.902-6.681)
**rs3847859 recessive**	AA	47	13.5	7	29.2	Ref.	1.000
	AG+GG	300	86.5	17	70.8	0.012	4.115 (1.364-12.413)

Table S102. Distribution of ALK1 rs3847859 genotypes according to the presence of pathological kidney disease (evaluated by CKD-EPI)

SNP	Genotype	Normal		Pathological		P value	OR (95% CI)
		N	%	N	%		
**rs3847859**	GG	55	32.2	65	31.3	Ref.	1.000
	AG	89	52.0	114	54.8	0.674	1.159 (0.584-2.298)
	AA	27	15.8	29	13.9	0.614	1.178 (0.623-2.228)
**rs3847859 dominant**	GG	55	32.2	65	31.2	Ref.	1.000
	AG+AA	116	67.8	143	68.8	0.946	0.983 (0.604-1.600)
**rs3847859 recessive**	AA	27	15.8	29	13.9	Ref.	1.000
	AG+GG	144	84.2	179	86.1	0.614	0.849 (0.449-1.605)

HEART RATE

Table S103. Distribution of ALK1 rs3847859 genotypes according to the values of heart rate (cutpoint: 100 bpm)

SNP	Genotype	< 100		> 100		P value	OR (95% CI)
		N	%	N	%		
**rs3847859**	GG	119	31.6	1	33.3	Ref.	1.000
	AG	202	53.7	1	33.3	0.571	0.446 (0.027-7.295)
	AA	55	14.7	1	33.3	0.364	0.275 (0.017-4.475)
**rs3847859 dominant**	GG	119	31.6	1	33.3	Ref.	1.000
	AG+AA	257	68.4	2	66.7	0.734	1.622 (0.100-26.371)
**rs3847859 recessive**	AA	55	14.6	1	33.3	Ref.	1.000
	AG+GG	321	85.4	2	66.7	0.364	3.639 (0.223-59.251)

Table S104. Distribution of ALK1 rs3847859 genotypes according to the values of heart rate (cutpoint: 90 bpm)

SNP	Genotype	< 90		> 90		P value	OR (95% CI)
		N	%	N	%		
**rs3847859**	GG	117	31.7	3	30.0	Ref.	1.000
	AG	198	53.7	5	50.0	0.699	0.699 (0.113-4.313)

	AA	54	14.6	2	20.0	0.649	0.679 (0.128-3.598)
**rs3847859 dominant**	GG	117	31.7	3	30.0	Ref.	1.000
	AG+AA	252	68.3	7	70.0	0.969	1.029 (0.240-4.407)
**rs3847859 recessive**	AA	54	14.6	2	20.0	Ref.	1.000
	AG+GG	315	85.4	8	80.0	0.649	1.473 (0.278-7.810)

Table S105. Distribution of ALK1 rs3847859 genotypes according to the values of heart rate (cutpoint: 70 bpm)

**SNP**	**Genotype**	**< 70**		**> 70**		**P value**	**OR (95% CI)**
		N	%	N	%		
**rs3847859**	GG	77	36.0	43	26.1	Ref.	1.000
	AG	115	53.7	88	53.3	0.002	0.362 (0.188-0.698)
	AA	22	10.3	34	20.6	0.023	0.495 (0.270-0.908)
**rs3847859 dominant**	GG	77	36.0	43	26.1	Ref.	1.000
	AG+AA	137	64.0	122	73.9	0.042	0.628 (0.401-0.984)
**rs3847859 recessive**	AA	22	10.3	34	20.6	Ref.	1.000
	AG+GG	192	89.7	131	79.4	0.006	2.263 (1.263-4.054)

Table S106. Distribution of ALK1 rs3847859 genotypes according to the values of heart rate (cutpoint: 50 bpm)

**SNP**	**Genotype**	**< 50**		**> 50**		**P value**	**OR (95% CI)**
		N	%	N	%		
**rs3847859**	GG	3	75.0	117	31.2	Ref.	1.000
	AG	1	25.0	202	53.9	0.997	0.000 (0.000-)
	AA	-	-	56	14.9	-	-
**rs3847859 dominant**	GG	3	75.0	117	31.2	Ref.	1.000
	AG+AA	1	25.0	258	68.8	0.191	0.217 (0.022-2.141)
**rs3847859 recessive**	AA	-	-	56	14.9	Ref.	1.000
	AG+GG	4	100.0	319	85.1	0.997	16488753.8 (0.000-)

3. Endoglin rs3739817 polymorphism

MEDICAL RECORD

Table S107. Distribution of endoglin rs3739817 genotypes according to the presence of previous history of cardiovascular disease

SNP	Genotype	No		Yes		P value	OR (95% CI)
		N	%	N	%		
**rs3739817**	GG	320	88.4	16	94.1	Ref.	1.000
	AG	40	11.0	1	5.9	0.999	726667886.1(0.000-)
	AA	2	0.6	-	-	-	-
**rs3739817 dominant**	GG	320	88.4	16	94.1	Ref.	1.000
	AG+AA	42	11.6	1	5.9	0.468	2.141 (0.274-16.708)
**rs3739817 recessive**	AA	2	0.6	-	-	Ref.	1.000
	AG+GG	360	99.4	17	100.0	1.000	0.000 (0.000-)

Table S108. Distribution of endoglin rs3739817 genotypes according to the presence of previous history of premature cardiovascular disease

SNP	Genotype	No		Yes		P value	OR (95% CI)
		N	%	N	%		
**rs3739817**	GG	320	88.4	16	94.1	Ref.	1.000
	AG	40	11.0	1	5.9	0.999	72666886.1 (0.000-)
	AA	2	0.6	-	-	-	-
**rs3739817 dominant**	GG	320	88.4	16	94.1	Ref.	1.000
	AG+AA	42	11.6	1	5.9	0.468	2.141 (0.274-16.708)
**rs3739817 recessive**	AA	2	0.6	-	-	Ref.	1.000
	AG+GG	360	99.4	17	100.0	1.000	0.000 (0.000-)

Table S109. Distribution of endoglin rs3739817 genotypes according to the presence of previous history of cerebrovascular disease

SNP	Genotype	No		Yes		P value	OR (95% CI)
		N	%	N	%		
**rs3739817**	GG	335	89.1	1	33.3	Ref.	1.000
	AG	39	10.4	2	66.7	1.000	3311610.38 (0.000-)
	AA	2	0.5	-	-	-	-
**rs3739817 dominant**	GG	335	89.1	1	33.3	Ref.	1.000
	AG+AA	41	10.9	2	66.7	0.021	0.055 (0.005-0.644)
**rs3739817 recessive**	AA	2	0.5	-	-	Ref.	1.000
	AG+GG	374	99.5	3	100.0	0.999	0.000 (0.000-)

Table S110. Distribution of endoglin rs3739817 genotypes according to the presence of previous history of heart failure

SNP	Genotype	No		Yes		P value	OR (95% CI)
		N	%	N	%		
**rs3739817**	GG	327	88.7	9	90.0	Ref.	1.000
	AG	40	10.8	1	10.0	1.000	27080727.4 (0.000-)
	AA	2	0.5	-	-	-	-
**rs3739817 dominant**	GG	327	88.6	9	90.0	Ref.	1.000
	AG+AA	42	11.4	1	10.0	0.831	1.261 (0.151-10.540)
**rs3739817 recessive**	AA	2	0.5	-	-	Ref.	1.000
	AG+GG	367	99.5	10	100.0	1.000	0.000 (0.000-)

Table S111. Distribution of endoglin rs3739817 genotypes according to the presence of previous personal history of dislipemia

SNP	Genotype	No		Yes		P value	OR (95% CI)
		N	%	N	%		
**rs3739817**	GG	107	89.2	229	88.4	Ref.	1.000
	AG	12	10.0	29	11.2	0.593	2.140 (0.132-34.729)
	AA	1	0.8	1	0.4	0.562	2.329 (0.133-40.676)
**rs3739817 dominant**	GG	107	89.2	229	88.4	Ref.	1.000
	AG+AA	13	10.8	30	11.6	0.818	0.919 (0.446-1.893)
**rs3739817 recessive**	AA	1	0.8	1	0.4	Ref.	1.000
	AG+GG	119	99.2	258	99.6	0.562	0.429 (0.025-7.498)

Table S112. Distribution of endoglin rs3739817 genotypes according to the presence of previous personal history of diabetes.

SNP	Genotype	No		Yes		P value	OR (95% CI)
		N	%	N	%		
**rs3739817**	GG	258	88.7	78	88.6	Ref.	1.000
	AG	31	10.7	10	11.4	0.999	471244685 (0.000-)
	AA	2	0.7	-	-	-	-
**rs3739817 dominant**	GG	258	88.7	78	88.6	Ref.	1.000
	AG+AA	33	11.3	10	11.4	0.901	0.952 (0.441-2.056)
**rs3739817 recessive**	AA	2	0.7	-	-	Ref.	1.000
	AG+GG	289	99.3	88	100.0	0.999	0.000 (0.000-)

Table S113. Distribution of endoglin rs3739817 genotypes according to the presence of previous personal history of hypertension.

SNP	Genotype	No		Yes		P value	OR (95% CI)
		N	%	N	%		
**rs3739817**	GG	90	88.2	246	88.8	Ref.	1.000
	AG	11	10.8	30	10.8	0.479	2.766 (0.165-46.308)
	AA	1	1.0	1	0.4	0.508	2.657 (0.147-48.040)
**rs3739817 dominant**	GG	90	88.2	246	88.8	Ref.	1.000
	AG+AA	12	11.8	31	11.2	0.916	1.041 (0.491-2.206)
**rs3739817 recessive**	AA	1	1.0	1	0.4	Ref.	1.000
	AG+GG	101	99.0	276	99.6	0.508	0.376 (0.021-6.805)

Table S114. Distribution of endoglin rs3739817 genotypes according to the presence of total peripheral arterial disease

SNP	Genotype	No		Yes		P value	OR (95% CI)
		N	%	N	%		
**rs3739817**	GG	309	88.8	5	100.0	Ref.	1.000
	AG	37	10.6	-	-	-	-
	AA	2	0.6	-	-	-	-
**rs3739817 dominant**	GG	309	88.8	5	100.0	Ref.	1.000
	AG+AA	39	11.2	-	-	-	-
**rs3739817 recessive**	AA	2	0.6	-	-	Ref.	1.000
	AG+GG	346	99.4	5	100.0	1.000	1.315 (0.000-)

Table S115. Distribution of endoglin rs3739817 genotypes according to the presence of left ventricular hypertrophy (evaluated by electrocardiography)

SNP	Genotype	No		Yes		P value	OR (95% CI)
		N	%	N	%		
**rs3739817**	GG	304	88.9	26	86.7	Ref.	1.000

	AG	36	10.5	4	13.3	0.999	137479028 (0.000-)
	AA	2	0.6	-	-	-	-
**rs3739817 dominant**	GG	304	88.9	26	86.7	Ref.	1.000
	AG+AA	38	11.1	4	13.3	0.622	0.756 (0.249-2.295)
**rs3739817 recessive**	AA	2	0.6	-	-	Ref.	1.000
	AG+GG	340	99.4	30	100.0	0.999	0.000 (0.000-)

Table S116. Distribution of endoglin rs3739817 genotypes according to the presence of left ventricular hypertrophy (evaluated by Cornell voltage duration product)

SNP	Genotype	No		Yes		P value	OR (95% CI)
		N	%	N	%		
**rs3739817**	GG	308	88.3	22	95.7	Ref.	1.000
	AG	39	11.1	1	4.3	0.999	106970374 (0.000-)
	AA	2	0.6	-	-	-	-
**rs3739817 dominant**	GG	308	88.3	22	95.7	Ref.	1.000
	AG+AA	41	11.7	1	4.3	0.271	3.171 (0.406-24.794)
**rs3739817 recessive**	AA	2	0.6	-	-	Ref.	1.000
	AG+GG	347	99.4	23	100.0	0.999	0.000 (0.000-)

Table S117. Distribution of endoglin rs3739817 genotypes according to the presence of left ventricular hypertrophy (evaluated by Sokolow-Lyon index)

SNP	Genotype	No		Yes		P value	OR (95% CI)
		N	%	N	%		
**rs3739817**	GG	330	88.5	5	100.0	Ref.	1.000
	AG	41	11.0	-	-	-	-
	AA	2	0.5	-	-	-	-
**rs3739817 dominant**	GG	330	88.5	5	100.0	Ref.	1.000
	AG+AA	43	11.5	-	-	-	-
**rs3739817 recessive**	AA	2	0.5	-	-	Ref.	1.000
	AG+GG	371	99.5	5	100.0	1.000	1.008 (0.000-)

Table S118. Distribution of endoglin rs3739817 genotypes according to the presence of left ventricular hypertrophy (evaluated by Lewis index)

SNP	Genotype	No		Yes		P value	OR (95% CI)
		N	%	N	%		
**rs3739817**	GG	204	88.3	31	91.2	Ref.	1.000
	AG	25	10.8	3	8.8	0.999	234292504 (0.000-)
	AA	2	0.9	-	-	0.999	174117317 (0.000-)
**rs3739817 dominant**	GG	204	88.3	31	91.2	Ref.	1.000
	AG+AA	27	11.7	3	8.8	0.983	0.976 (0.107-8.865)
**rs3739817 recessive**	AA	2	0.9	-	-	Ref.	1.000
	AG+GG	229	99.1	34	100.0	0.209	2.906 (0.551-15.318)

Table S119. Distribution of endoglin rs3739817 genotypes according to the presence of abdominal obesity

SNP	Genotype	No		Yes		P value	OR (95% CI)
		N	%	N	%		
**rs3739817**	GG	161	91.5	175	86.2	Ref.	1.000
	AG	13	7.4	28	13.8	0.999	1.857E+9 (0.000-)
	AA	2	1.1	-	-	-	-
**rs3739817 dominant**	GG	161	91.5	175	86.2	Ref.	1.000
	AG+AA	15	8.5	28	13.8	0.057	0.505 (0.250-1.019)

**rs3739817 recessive**	AA	2	1.1	-	-	Ref.	1.000
	AG+GG	174	98.9	203	100.0	0.999	0.000 (0.000-)

Table S120. Distribution of endoglin rs3739817 genotypes according to the presence of metabolic syndrome

SNP	Genotype	No		Yes		P value	OR (95% CI)
		N	%	N	%		
**rs3739817**	GG	250	87.7	86	91.5	Ref.	1.000
	AG	33	11.6	8	8.5	0.999	543809408 (0.000-)
	AA	2	0.7	-	-	-	-
**rs3739817 dominant**	GG	250	87.7	86	91.5	Ref.	1.000
	AG+AA	35	12.3	8	8.5	0.441	1.387 (0.603-3.186)
**rs3739817 recessive**	AA	2	0.7	-	-	Ref.	1.000
	AG+GG	283	99.3	94	100.0	0.999	0.000 (0.000-)

ANALYTICAL VALUES

Table S121. Distribution of endoglin rs3739817 genotypes according to the presence of elevated cholesterol plasma levels

SNP	Genotype	No		Yes		P value	OR (95% CI)
		N	%	N	%		
**rs3739817**	GG	104	91.2	219	86.9	Ref.	1.000
	AG	10	8.8	31	12.3	0.999	0.000 (0.000-)
	AA	-	-	2	0.8	-	-
**rs3739817 dominant**	GG	104	91.2	219	86.9	Ref.	1.000
	AG+AA	10	8.8	33	13.1	0.277	0.657 (0.308-1.400)
**rs3739817 recessive**	AA	-	-	2	0.8	Ref.	1.000
	AG+GG	114	100.0	250	99.2	0.999	486543803 (0.000-)

Table S122. Distribution of endoglin rs3739817 genotypes according to the presence of reduced cholesterol-high density lipoprotein plasma levels

SNP	Genotype	No		Yes		P value	OR (95% CI)
		N	%	N	%		
**rs3739817**	GG	237	86.8	86	92.5	Ref.	1.000
	AG	34	12.5	7	7.5	0.999	622509467 (0.000-)
	AA	2	0.7	-	-	-	-
**rs3739817 dominant**	GG	237	86.8	86	92.5	Ref.	1.000
	AG+AA	36	13.2	7	7.5	0.150	1.898 (0.793-4.543)
**rs3739817 recessive**	AA	2	0.7	-	-	Ref.	1.000
	AG+GG	271	99.3	93	100.0	0.999	0.000 (0.000-)

Table S123. Distribution of endoglin rs3739817 genotypes according to the presence of elevated cholesterol-low density lipoprotein plasma levels

SNP	Genotype	No		Yes		P value	OR (95% CI)
		N	%	N	%		
**rs3739817**	GG	98	86.0	233	90.0	Ref.	1.000
	AG	16	14.0	24	9.2	0.999	0.000 (0.000-)
	AA	-	-	2	0.8	0.999	0.000 (0.000-)
**rs3739817 dominant**	GG	98	86.0	233	90.0	Ref.	1.000
	AG+AA	16	14.0	26	10.0	0.229	1.526 (0.766-3.042)
**rs3739817 recessive**	AA	-	-	2	0.8	Ref.	1.000
	AG+GG	114	100.0	257	99.2	0.999	978176857 (0.000-)

Table S124. Distribution of endoglin rs3739817 genotypes according to the presence of elevated triglycerides plasma levels

SNP	Genotype	No		Yes		P value	OR (95% CI)
		N	%	N	%		
rs3739817	GG	249	88.0	87	90.6	Ref.	1.000
	AG	32	11.3	9	9.4	0.999	554771591 (0.000-)
	AA	2	0.7	-	-	-	-
rs3739817 dominant	GG	249	88.0	87	90.6	Ref.	1.000
	AG+AA	34	12.0	9	9.4	0.642	1.205 (0.548-2.647)
rs3739817 recessive	AA	2	0.7	-	-	Ref.	1.000
	AG+GG	281	99.3	96	100.0	0.999	0.000 (0.000-)

Table S125. Distribution of endoglin rs3739817 genotypes according to the presence of dislipemia

SNP	Genotype	No		Yes		P value	OR (95% CI)
		N	%	N	%		
rs3739817	GG	20	90.9	315	88.4	Ref.	1.000
	AG	2	9.1	39	11.0	0.999	0.000 (0.000-)
	AA	-	-	2	0.6	-	-
rs3739817 dominant	GG	20	90.9	315	88.5	Ref.	1.000
	AG+AA	2	9.1	41	11.5	0.701	0.742 (0.162-3.402)
rs3739817 recessive	AA	-	-	2	0.6	Ref.	1.000
	AG+GG	22	100.0	354	99.4	0.999	56554638.2 (0.000-)

Table S126. Distribution of endoglin rs3739817 genotypes according to the presence of altered basal glycemia

SNP	Genotype	No		Yes		P value	OR (95% CI)
		N	%	N	%		
rs3739817	GG	296	88.6	40	88.9	Ref.	1.000
	AG	36	10.8	5	11.1	0.999	215110147 (0.000-)
	AA	2	0.6	-	-	-	-
rs3739817 dominant	GG	296	88.6	40	88.9	Ref.	1.000
	AG+AA	38	11.4	5	11.1	0.992	0.995 (0.368-2.691)
rs3739817 recessive	AA	2	0.6	-	-	Ref.	1.000
	AG+GG	332	99.4	45	100.0	0.999	0.000 (0.000-)

Table S127. Distribution of endoglin rs3739817 genotypes according to the presence of microalbuminuria

SNP	Genotype	No		Yes		P value	OR (95% CI)
		N	%	N	%		
rs3739817	GG	309	88.0	20	100.0	Ref.	1.000
	AG	40	11.4	-	-	-	-
	AA	2	0.6	-	-	-	-
rs3739817 dominant	GG	309	88.0	20	100.0	Ref.	1.000
	AG+AA	42	12.0	-	-	-	-
rs3739817 recessive	AA	2	0.6	-	-	Ref.	1.000
	AG+GG	349	99.4	20	100.0	1.000	1.033 (0.000-)

CARDIOVASCULAR TESTS

Table S128. Distribution of endoglin rs3739817 genotypes according to the presence of elevated pulse pressure

SNP	Genotype	No		Yes		P value	OR (95% CI)
		N	%	N	%		
**rs3739817**	GG	257	89.5	79	85.9	Ref.	1.000
	AG	29	10.2	12	13.0	0.324	0.241 (0.014-4.062)
	AA	1	0.3	1	1.1	0.421	0.304 (0.017-5.537)
**rs3739817 dominant**	GG	257	89.5	79	85.9	Ref.	1.000
	AG+AA	30	10.5	13	14.1	0.552	0.793 (0.370-1.701)
**rs3739817 recessive**	AA	1	0.3	1	1.1	Ref.	1.000
	AG+GG	286	99.7	91	98.9	0.421	3.290 (0.181-59.921)

Table S129. Distribution of endoglin rs3739817 genotypes according to the presence of increased carotid intima-media thickness

SNP	Genotype	No		Yes		P value	OR (95% CI)
		N	%	N	%		
**rs3739817**	GG	276	89.1	59	88.1	Ref.	1.000
	AG	32	10.3	8	11.9	0.999	318003694 (0.000-)
	AA	2	0.6	-	-	-	-
**rs3739817 dominant**	GG	276	89.0	59	88.1	Ref.	1.000
	AG+AA	34	11.0	8	11.9	0.741	0.867 (0.372-2.021)
**rs3739817 recessive**	AA	2	0.6	-	-	Ref.	1.000
	AG+GG	308	99.4	67	100.0	0.999	0.000 (0.000-)

Table S130. Distribution of endoglin rs3739817 genotypes according to the values of pulse wave velocity

SNP	Genotype	No		Yes		P value	OR (95% CI)
		N	%	N	%		
**rs3739817**	GG	283	89.3	48	84.2	Ref.	1.000
	AG	32	10.1	9	15.8	0.999	58389205.1 (0.000-)
	AA	2	0.6	-	-	-	-
**rs3739817 dominant**	GG	283	89.3	48	84.2	Ref.	1.000
	AG+AA	34	10.7	9	15.8	0.549	0.732 (0.263-2.031)
**rs3739817 recessive**	AA	2	0.6	-	-	Ref.	1.000
	AG+GG	315	99.4	57	100.	0.999	0.000 (0.000-)

Table S131. Distribution of endoglin rs3739817 genotypes according to the presence of pathological ankle-brachial index

SNP	Genotype	No		Yes		P value	OR (95% CI)
		N	%	N	%		
**rs3739817**	GG	330	88.5	5	100.0	Ref.	1.000
	AG	41	11.0	-	-	-	-
	AA	2	0.5	-	-	-	-
**rs3739817 dominant**	GG	330	88.5	5	100.0	Ref.	1.000
	AG+AA	43	11.5	-	-	-	-
**rs3739817 recessive**	AA	2	0.5	-	-	Ref.	1.000
	AG+GG	371	99.5	5	100.0	1.000	1.294 (0.000-)

CURRENT DISEASES

Table S132. Distribution of endoglin rs3739817 genotypes according to the presence of cerebrovascular disease

SNP	Genotype	No		Yes		P value	OR (95% CI)
		N	%	N	%		
**rs3739817**	GG	335	89.1	1	33.3	Ref.	1.000
	AG	39	10.4	2	66.7	1.000	3311610.38 (0.000-)
	AA	2	0.5	-	-	-	-
**rs3739817 dominant**	GG	335	89.1	1	33.3	Ref.	1.000
	AG+AA	41	10.9	2	66.7	0.021	0.055 (0.005-0.644)
**rs3739817 recessive**	AA	2	0.5	-	-	Ref.	1.000
	AG+GG	374	99.5	3	100.0	0.999	0.000 (0.000-)

Table S133. Distribution of endoglin rs3739817 genotypes according to the presence of cardiovascular disease

SNP	Genotype	No		Yes		P value	OR (95% CI)
		N	%	N	%		
**rs3739817**	GG	327	88.6	9	90.0	Ref.	1.000
	AG	40	10.9	1	10.0	1.000	27080727.4 (0.000-)
	AA	2	0.5	-	-	-	-
**rs3739817 dominant**	GG	327	88.6	9	90.0	Ref.	1.000
	AG+AA	42	11.4	1	10.0	0.831	1.261 (0.151-10.540)
**rs3739817 recessive**	AA	2	0.5	-	-	Ref.	1.000
	AG+GG	367	99.5	10	100.0	1.000	0.000 (0.000-.)

Table S134. Distribution of endoglin rs3739817 genotypes according to the presence of kidney disease

SNP	Genotype	No		Yes		P value	OR (95% CI)
		N	%	N	%		
**rs3739817**	GG	325	88.6	4	100.0	Ref.	1.000
	AG	40	10.9	-	-	-	-
	AA	2	0.5	-	-	-	-
**rs3739817 dominant**	GG	325	88.6	4	100.0	Ref.	1.000
	AG+AA	42	11.4	-	-	-	-
**rs3739817 recessive**	AA	2	0.5	-	-	Ref.	1.000
	AG+GG	365	99.5	4	100.0	1.000	1.294 (0.000-)

Table S135. Distribution of endoglin rs3739817 genotypes according to the presence of advanced retinopathy

SNP	Genotype	No		Yes		P value	OR (95% CI)
		N	%	N	%		
**rs3739817**	GG	114	88.4	7	87.5	Ref.	1.000
	AG	15	11.6	1	12.5	0.983	0.976 (0.107-8.865)
	AA	-	-	-	-	-	-
**rs3739817 dominant**	GG	114	88.4	7	87.5	Ref.	1.000
	AG+AA	15	11.6	1	12.5	0.983	0.976 (0.107-8.865)
**rs3739817 recessive**	AA	-	-	-	-	Ref.	1.000
	AG+GG	129	100.0	8	100.0	1.000	1.192 (0.000-)

Table S136. Distribution of endoglin rs3739817 genotypes according to the presence of controlled arterial pressure.

SNP	Genotype	< average		> average		P value	OR (95% CI)
		N	%	N	%		

**rs3739817**	GG	45	81.8	38	92.7	Ref.	1.000
	AG	10	18.2	3	7.3	0.079	3.523 (0.864-14.357)
	AA	-	-	-	-	-	-
**rs3739817 dominant**	GG	45	81.8	38	92.7	Ref.	1.000
	AG+AA	10	18.2	3	7.3	0.079	3.523 (0.326-14.357)
**rs3739817 recessive**	AA	-	-	-	-	Ref.	1.000
	AG+GG	55	100.0	41	100.0	0.527	1.320 (0.559-3.118)

Table S137. Distribution of endoglin rs3739817 genotypes according to the presence of target organ damage

SNP	Genotype	No		Yes		P value	OR (95% CI)
		N	%	N	%		
**rs3739817**	GG	52	86.7	29	85.3	Ref.	1.000
	AG	8	13.3	5	14.7	0.946	0.958 (0.281-3.270)
	AA	-	-	-	-	-	-
**rs3739817 dominant**	GG	52	86.7	29	85.3	Ref.	1.000
	AG+AA	8	13.3	5	14.7	0.946	0.958 (0.281-3.270)
**rs3739817 recessive**	AA	-	-	-	-	Ref.	1.000
	AG+GG	60	100.0	34	100.0	0.899	1.058 (0.444-2.522)

Table S138. Distribution of endoglin rs3739817 genotypes according to the presence of hypertension, diabetes and obesity

SNP	Genotype	No		Yes		P value	OR (95% CI)
		N	%	N	%		
**rs3739817**	GG	107	88.4	229	88.8	Ref.	1.000
	AG	13	10.8	28	10.8	0.600	2.120 (0.128-35.149)
	AA	1	0.8	1	0.4	0.608	2.123 (0.120-37.684)
**rs3739817 dominant**	GG	107	88.4	229	88.8	Ref.	1.000
	AG+AA	14	11.6	29	11.2	0.997	0.999 (0.494-2.018)
**rs3739817 recessive**	AA	1	0.8	1	0.4	Ref.	1.000
	AG+GG	120	99.2	157	99.6	0.608	0.471 (0.027-8.359)

Table S139. Distribution of endoglin rs3739817 genotypes according to the presence of hypertension and diabetes

SNP	Genotype	No		Yes		P value	OR (95% CI)
		N	%	N	%		
**rs3739817**	GG	142	89.9	194	87.8	Ref.	1.000
	AG	15	9.5	26	11.7	0.840	1.338 (0.079-22.599)
	AA	1	0.6	1	0.5	0.720	1.698 (0.094-30.600)
**rs3739817 dominant**	GG	142	89.9	194	87.8	Ref.	1.000
	AG+AA	16	10.1	27	12.2	0.494	0.788 (0.398-1.560)
**rs3739817 recessive**	AA	1	0.6	1	0.5	Ref.	1.000
	AG+GG	157	99.4	220	99.5	0.720	0.589 (0.033-10.616)

RISK SCALES

Table S140. Distribution of endoglin rs3739817 genotypes according to the presence of cardiovascular risk

SNP	Genotype	Low		High		P value	OR (95% CI)
		N	%	N	%		
**rs3739817**	GG	28	90.3	295	88.1	Ref.	1.000
	AG	3	9.7	38	11.3	0.999	0.000 (0.000-)

	AA	-	-	2	0.6	0.999	0.000 (0.000-)
**rs3739817 dominant**	GG	28	90.3	295	88.1	Ref.	1.000
	AG+AA	3	9.7	40	11.9	0.789	0.825 (0.202-3.366)
**rs3739817 recessive**	AA	-	-	2	0.6	Ref.	1.000
	AG+GG	31	100.0	333	99.4	0.999	92086215.1 (0.000-)

Table S141. Distribution of endoglin rs3739817 genotypes according to the presence of diabetes (based on 2013 guidelines)

SNP	Genotype	No		Yes		P value	OR (95% CI)
		N	%	N	%		
**rs3739817**	GG	282	89.2	54	85.7	Ref.	1.000
	AG	32	10.2	9	14.3	0.999	294778875 (0.000-)
	AA	2	0.6	-	-	-	-
**rs3739817 dominant**	GG	282	89.2	54	85.7	Ref.	1.000
	AG+AA	34	10.8	9	14.3	0.358	0.684 (0.305-1.537)
**rs3739817 recessive**	AA	2	0.6	-	-	Ref.	1.000
	AG+GG	314	99.4	63	100.0	0.999	0.000 (0.000-)

Table S142. Distribution of endoglin rs3739817 genotypes according to the presence of hypertension (based on 2013 guidelines)

SNP	Genotype	No		Yes		P value	OR (95% CI)
		N	%	N	%		
**rs3739817**	GG	175	89.7	161	87.5	Ref.	1.000
	AG	19	9.8	22	12.0	0.939	0.897 (0.054-14.797)
	AA	1	0.5	1	0.5	0.937	1.123 (0.064-19.664)
**rs3739817 dominant**	GG	175	89.7	161	87.5	Ref.	1.000
	AG+AA	20	10.3	23	12.5	0.503	0.799 (0.414-1.541)
**rs3739817 recessive**	AA	1	0.5	1	0.5	Ref.	1.000
	AG+GG	194	99.5	183	99.5	0.937	0.891 (0.051-15.596)

RETINOPATHY

Table S143. Distribution of endoglin rs3739817 genotypes according to the caliber of medium retinal arteries.

SNP	Genotype	< average		> average		P value	OR (95% CI)
		N	%	N	%		
**rs3739817**	GG	110	88.0	99	87.6	Ref.	1.000
	AG	15	12.0	14	12.4	0.908	0.955 (0.437-2.087)
	AA	-	-	-	-	-	-
**rs3739817 dominant**	GG	110	88.0	99	87.6	Ref.	1.000
	AG+AA	15	12.0	14	12.4	0.908	0.955 (0.437-2.087)
**rs3739817 recessive**	AA	-	-	-	-	Ref.	1.000
	AG+GG	125	100.0	113	100.0	0.511	0.841 (0.503-1.408)

Table S144. Distribution of endoglin rs3739817 genotypes according to the caliber of right retinal arteries.

SNP	Genotype	< average		> average		P value	OR (95% CI)
		N	%	N	%		
**rs3739817**	GG	94	87.0	92	88.5	Ref.	1.000
	AG	14	13.0	12	11.5	0.775	1.128 (0.493-2.580)
	AA	-	-	-	-	-	-
	GG	94	87.0	92	88.5	Ref.	1.000

**rs3739817 dominant**	AG+AA	14	13.0	12	11.5	0.775	1.128 (0.493-2.580)
**rs3739817 recessive**	AA	-	-	-	-	Ref.	1.000
	AG+GG	108	100.0	104	100.0	0.258	1.371 (0.794-2.368)

Table S145. Distribution of endoglin rs3739817 genotypes according to the caliber of minor retinal arteries.

SNP	Genotype	< average		> average		P value	OR (95% CI)
		N	%	N	%		
**rs3739817**	GG	109	88.6	100	87.0	Ref.	1.000
	AG	14	11.4	15	13.0	0.614	0.817 (0.373-1.789)
	AA	-	-	-	-	-	-
**rs3739817 dominant**	GG	109	88.6	100	87.0	Ref.	1.000
	AG+AA	14	11.3	15	13.0	0.614	0.817 (0.373-1.789)
**rs3739817 recessive**	AA	-	-	-	-	Ref.	1.000
	AG+GG	123	100.0	115	100.0	0.256	1.349 (0.805-2.260)

Table S146. Distribution of endoglin rs3739817 genotypes according to the caliber of left retinal arteries.

SNP	Genotype	< average		> average		P value	OR (95% CI)
		N	%	N	%		
**rs3739817**	GG	95	91.3	90	84.1	Ref.	1.000
	AG	9	8.7	17	15.9	0.109	0.489 (0.204-1.172)
	AA	-	-	-	-	-	-
**rs3739817 dominant**	GG	95	91.3	90	84.1	Ref.	1.000
	AG+AA	9	8.7	17	15.9	0.109	2.150 (1.028-1.172)
**rs3739817 recessive**	AA	-	-	-	-	Ref.	1.000
	AG+GG	104	100.0	107	100.0	0.050	0.573 (0.328-1.001)

Table S147. Distribution of endoglin rs3739817 genotypes according to the values of left arteriovenous index (AVIx)

SNP	Genotype	< average		> average		P value	OR (95% CI)
		N	%	N	%		
**rs3739817**	GG	74	88.1	79	85.9	Ref.	1.000
	AG	10	11.9	13	14.1	0.664	0.819 (0.334-2.010)
	AA	-	-	-	-	-	-
**rs3739817 dominant**	GG	74	88.1	79	85.9	Ref.	1.000
	AG+AA	10	11.9	13	14.1	0.664	0.819 (0.334-2.010)
**rs3739817 recessive**	AA	-	-	-	-	Ref.	1.000
	AG+GG	84	100.0	92	100.0	0.132	0.628 (0.343-1.151)

Table S148. Distribution of endoglin rs3739817 genotypes according to the values of right arteriovenous index (AVIx)

SNP	Genotype	< average		> average		P value	OR (95% CI)
		N	%	N	%		
**rs3739817**	GG	74	90.2	67	84.8	Ref.	1.000
	AG	8	9.8	12	15.2	0.322	0.616 (0.236-1.606)
	AA	-	-	-	-	-	-
**rs3739817 dominant**	GG	74	90.2	67	84.8	Ref.	1.000
	AG+AA	8	9.8	12	15.2	0.322	0.616 (0.236-1.606)
**rs3739817 recessive**	AA	-	-	-	-	Ref.	1.000
	AG+GG	82	100.0	79	100.0	0.528	0.818 (0.437-1.529)

Table S149. Distribution of endoglin rs3739817 genotypes according to the values of medium arteriovenous index (AVIx)

SNP	Genotype	< average		> average		P value	OR (95% CI)
		N	%	N	%		
rs3739817	GG	113	91.9	94	83.2	Ref.	1.000
	AG	10	8.1	19	16.8	0.046	0.435 (0.192-0.986)
	AA	-	-	-	-	-	-
rs3739817 dominant	GG	113	91.9	94	83.2	Ref.	1.000
	AG+AA	10	8.1	19	16.8	0.046	0.435 (0.192-0.986)
rs3739817 recessive	AA	-	-	-	-	Ref.	1.000
	AG+GG	123	100.0	113	100.0	0.325	0.769 (0.456-1.297)

Table S150. Distribution of endoglin rs3739817 genotypes according to the caliber of left retinal veins

SNP	Genotype	< average		> average		P value	OR (95% CI)
		N	%	N	%		
rs3739817	GG	97	84.3	88	91.7	Ref.	1.000
	AG	18	15.7	8	8.3	0.105	2.083 (0.859-5.056)
	AA	-	-	-	-	-	-
rs3739817 dominant	GG	97	84.3	88	91.7	Ref.	1.000
	AG+AA	18	15.7	8	8.3	0.105	2.083 (0.859-5.056)
rs3739817 recessive	AA	-	-	-	-	Ref.	1.000
	AG+GG	115	100.0	96	100.0	0.770	1.086 (0.625-1.887)

Table S151. Distribution of endoglin rs3739817 genotypes according to the caliber of right retinal veins.

SNP	Genotype	< average		> average		P value	OR (95% CI)
		N	%	N	%		
rs3739817	GG	96	85.0	90	90.9	Ref.	1.000
	AG	17	15.0	9	9.1	0.202	1.751 (0.740-4.141)
	AA	-	-	-	-	-	-
rs3739817 dominant	GG	96	85.0	90	90.9	Ref.	1.000
	AG+AA	17	15.0	9	9.1	0.202	1.751 (0.740-4.141)
rs3739817 recessive	AA	-	-	-	-	Ref.	1.000
	AG+GG	113	100.0	99	100.0	0.309	1.330 (0.768-2.303)

Table S152. Distribution of endoglin rs3739817 genotypes according to the caliber of medium retinal veins.

SNP	Genotype	< average		> average		P value	OR (95% CI)
		N	%	N	%		
rs3739817	GG	113	85.0	96	91.4	Ref.	1.000
	AG	20	15.0	9	8.6	0.147	1.855 (0.805-4.277)
	AA	-	-	-	-	-	-
rs3739817 dominant	GG	113	85.0	96	91.4	Ref.	1.000
	AG+AA	20	15.0	9	8.6	0.147	1.855 (0.805-4.277)
rs3739817 recessive	AA	-	-	-	-	Ref.	1.000
	AG+GG	133	100.0	105	100.0	0.472	1.210 (0.719-2.037)

Table S153. Distribution of endoglin rs3739817 genotypes according to the caliber of major retinal veins.

SNP	Genotype	< average		> average		P value	OR (95% CI)
		N	%	N	%		
**rs3739817**	GG	105	84.7	104	91.2	Ref.	1.000
	AG	19	15.3	10	8.8	0.111	1.942 (0.858-4.393)
	AA	-	-	-	-	-	-
**rs3739817 dominant**	GG	105	84.7	104	91.2	Ref.	1.000
	AG+AA	19	15.3	10	8.8	0.111	1.942 (0.858-4.393)
**rs3739817 recessive**	AA	-	-	-	-	Ref.	1.000
	AG+GG	124	100.0	114	100.0	0.861	0.955 (0.569-1.602)

KIDNEY DISEASE

Table S154. Distribution of endoglin rs3739817 genotypes according to the presence of pathological albumin/creatinine index

SNP	Genotype	Normal		Pathological		P value	OR (95% CI)
		N	%	N	%		
**rs3739817**	GG	305	87.9	24	100.0	Ref.	1.000
	AG	40	11.5	-	-	-	-
	AA	2	0.6	-	-	-	-
**rs3739817 dominant**	GG	305	87.9	24	100.0	Ref.	1.000
	AG+AA	42	12.1	-	-	-	-
**rs3739817 recessive**	AA	2	0.6	-	-	Ref.	1.000
	AG+GG	345	99.4	24	100.0	1.000	1.055 (0.000-)

Table S155. Distribution of endoglin rs3739817 genotypes according to the presence of pathological kidney disease (evaluated by CKD-EPI)

SNP	Genotype	Normal		Pathological		P value	OR (95% CI)
		N	%	N	%		
**rs3739817**	GG	151	88.3	185	88.9	Ref.	1.000
	AG	20	11.7	21	10.1	0.999	0.000 (0.000-)
	AA	-	-	2	1.0	0.999	0.000 (0.000-)
**rs3739817 dominant**	GG	151	88.3	185	88.9	Ref.	1.000
	AG+AA	20	11.7	23	11.1	0.468	1.295 (0.644-2.603)
**rs3739817 recessive**	AA	-	-	2	1.0	Ref.	1.000
	AG+GG	171	100.0	206	99.0	0.999	1.776E+9 (0.000-)

HEART RATE

Table S156. Distribution of endoglin rs3739817 genotypes according to the values of heart rate (cutpoint: 100 bpm)

SNP	Genotype	< 100		> 100		P value	OR (95% CI)
		N	%	N	%		
**rs3739817**	GG	333	88.6	3	100.0	Ref.	1.000
	AG	41	10.9	-	-	-	-
	AA	2	0.5	-	-	-	-
**rs3739817 dominant**	GG	333	88.6	3	100.0	Ref.	1.000
	AG+AA	43	11.4	-	-	-	-
**rs3739817 recessive**	AA	2	0.5	-	-	Ref.	1.000
	AG+GG	374	99.5	3	100.0	1.000	1.003 (0.000-)

Table S157. Distribution of endoglin rs3739817 genotypes according to the values of heart rate (cutpoint: 90 bpm)

SNP	Genotype	< 90		> 90		P value	OR (95% CI)
		N	%	N	%		
**rs3739817**	GG	326	88.3	10	100.0	Ref.	1.000
	AG	41	11.2	-	-	-	-
	AA	2	0.5	-	-	1.000	0.991 (0.000-)
**rs3739817 dominant**	GG	326	88.3	10	100.0	Ref.	1.000
	AG+AA	43	11.7	-	-	-	-
**rs3739817 recessive**	AA	2	0.5	-	-	Ref.	1.000
	AG+GG	367	99.5	10	100.0	1.000	1.009 (0.000-)

Table S158. Distribution of endoglin rs3739817 genotypes according to the values of heart rate (cutpoint: 70 bpm)

SNP	Genotype	< 70		> 70		P value	OR (95% CI)
		N	%	N	%		
**rs3739817**	GG	184	86.0	152	92.1	Ref.	1.000
	AG	28	13.1	13	7.9	0.999	1.36E+9 (0.000-)
	AA	2	0.9	-	-	-	-
**rs3739817 dominant**	GG	184	86.0	152	92.1	Ref.	1.000
	AG+AA	30	14.0	13	7.9	0.107	1.770 (0.883-3.546)
**rs3739817 recessive**	AA	2	0.9	-	-	Ref.	1.000
	AG+GG	212	99.1	165	100.0	0.999	0.000 (0.000-)

Table S159. Distribution of endoglin rs3739817 genotypes according to the values of heart rate (cutpoint: 50 bpm)

SNP	Genotype	< 50		> 50		P value	OR (95% CI)
		N	%	N	%		
**rs3739817**	GG	4	100.0	332	88.6	Ref.	1.000
	AG	-	-	41	10.9	-	-
	AA	-	-	2	0.5	-	-
**rs3739817 dominant**	GG	4	100.0	332	88.5	Ref.	1.000
	AG+AA	-	-	43	11.5	-	-
**rs3739817 recessive**	AA	-	-	2	0.5	Ref.	1.000
	AG+GG	4	100.0	373	99.5	1.000	0.916 (0.000-)

4. Endoglin rs10987759 polymorphism

MEDICAL RECORD

Table S160. Distribution of endoglin rs10987759 genotypes according to the presence of previous history of cardiovascular disease

SNP	Genotype	No		Yes		P value	OR (95% CI)
		N	%	N	%		
**rs10987759**	TT	307	84.4	15	88.2	Ref.	1.000
	CT	54	14.9	2	11.8	1.000	912111569.8 (0.000-)
	CC	1	0.3	-	-	-	-
**rs10987759 dominant**	TT	307	84.8	15	88.2	Ref.	1.000
	CT+CC	55	15.2	2	11.8	0.313	1.820 (0.569-5.830)
**rs10987759 recessive**	CC	1	0.3	-	-	Ref.	1.000
	CT+TT	361	99.7	17	100.0	0.058	3.296 (0.960-11.311)

Table S161. Distribution of endoglin rs10987759 genotypes according to the presence of previous history of premature cardiovascular disease

SNP	Genotype	No		Yes		P value	OR (95% CI)
		N	%	N	%		
**rs10987759**	TT	307	84.8	15	88.2	Ref.	1.000
	CT	54	14.9	2	11.8	0.348	0.552 (0.160-1.910)
	CC	1	0.3	-	-	-	-
**rs10987759 dominant**	TT	307	84.8	15	88.2	Ref.	1.000
	CT+CC	55	15.2	2	11.8	1.000	91211569.8 (0.000-)
**rs10987759 recessive**	CC	1	0.3	-	-	Ref.	1.000
	CT+TT	361	99.7	17	100.0	1.000	75561722.3 (0.000-)

Table S162. Distribution of endoglin rs10987759 genotypes according to the presence of previous history of cerebrovascular disease

SNP	Genotype	No		Yes		P value	OR (95% CI)
		N	%	N	%		
**rs10987759**	TT	320	85.1	2	66.7	Ref.	1.000
	CT	55	14.6	1	33.3	1.000	0.861 (0.000-)
	CC	1	0.3	-	-	-	-
**rs10987759 dominant**	TT	320	85.1	2	66.7	Ref.	1.000
	CT+CC	56	14.9	1	33.3	0.800	0.730 (0.064-8.278)
**rs10987759 recessive**	CC	1	0.3	-	-	Ref.	1.000
	CT+TT	375	99.7	3	100.0	0.997	0.000 (0.000-)

Table S163. Distribution of endoglin rs10987759 genotypes according to the presence of previous history of heart failure

SNP	Genotype	No		Yes		P value	OR (95% CI)
		N	%	N	%		
**rs10987759**	TT	313	84.8	9	90.0	Ref.	1.000
	CT	55	14.9	1	10.0	1.000	124067944.2 (0.000-)
	CC	1	0.3	-	-	-	-
**rs10987759 dominant**	TT	313	84.8	9	90.0	Ref.	1.000
	CT+CC	56	15.2	1	10.0	0.156	2.905 (0.667-12.657)
**rs10987759 recessive**	CC	1	0.3	-	-	Ref.	1.000
	CT+TT	368	99.7	10	100.0	0.341	2.444 (0.389-15.364)

Table S164. Distribution of endoglin rs10987759 genotypes according to the presence of dislipemia

SNP	Genotype	No		Yes		P value	OR (95% CI)
		N	%	N	%		
**rs10987759**	TT	101	84.2	221	85.3	Ref.	1.000
	CT	18	15.0	38	14.7	1.000	3.803E+9 (0.000-)
	CC	1	0.8	-	-	-	-
**rs10987759 dominant**	TT	101	84.2	221	85.3	Ref.	1.000
	CT+CC	19	15.8	38	14.7	0.455	0.828 (0.506-1.357)
**rs10987759 recessive**	CC	1	0.8	-	-	Ref.	1.000
	CT+TT	119	99.46	84.5	100.0	0.584	0.836 (0.439-1.589)

Table S165. Distribution of endoglin rs10987759 genotypes according to the presence of previous personal history of diabetes.

SNP	Genotype	No		Yes		P value	OR (95% CI)
		N	%	N	%		
**rs10987759**	TT	246	84.5	76	86.4	Ref.	1.000
	CT	45	15.5	11	12.5	1.000	0.000 (0.000-)
	CC	-	-	1	1.1	-	-
**rs10987759 dominant**	TT	246	84.5	76	86.4	Ref.	1.000
	CT+CC	45	15.5	12	13.6	0.181	1.448 (0.842-2490)
**rs10987759 recessive**	CC	-	-	1	1.1	Ref.	1.000
	CT+TT	291	100.0	87	98.9	0.149	1.658 (0.834-3.298)

Table S166. Distribution of endoglin rs10987759 genotypes according to the presence of previous personal history of hypertension.

SNP	Genotype	No		Yes		P value	OR (95% CI)
		N	%	N	%		
**rs10987759**	TT	87	85.3	235	84.8	Ref.	1.000
	CT	14	13.7	42	15.2	1.000	4.014E+9 (0.000-)
	CC	1	1.0	-	-	-	-
**rs10987759 dominant**	TT	87	85.3	235	84.8	Ref.	1.000
	CT+CC	15	14.7	42	15.2	0.709	0.904 (0.532-1.536)
**rs10987759 recessive**	CC	1	1.0	-	-	Ref.	1.000
	CT+TT	101	99.0	277	100.0	0.112	0.589 (0.307-1.130)

Table S167. Distribution of endoglin rs10987759 genotypes according to the presence of total peripheral arterial disease

SNP	Genotype	No		Yes		P value	OR (95% CI)
		N	%	N	%		
**rs10987759**	TT	295	84.8	4	80.0	Ref.	1.000
	CT	52	14.9	1	20.0	1.000	0.964 (0.000-)
	CC	1	0.3	-	-	-	-
**rs10987759 dominant**	TT	295	84.8	4	80.0	Ref.	1.000
	CT+CC	53	15.2	1	20.0	0.528	0.477 (0.048-4.738)
**rs10987759 recessive**	CC	1	0.3	-	-	Ref.	1.000
	CT+TT	347	99.7	5	100.0	0.815	1.319 (0.129-13.536)

Table S168. Distribution of endoglin rs10987759 genotypes according to the presence of left ventricular hypertrophy (evaluated by electrocardiography)

SNP	Genotype	No		Yes		P value	OR (95% CI)
		N	%	N	%		
**rs10987759**	TT	294	86.0	23	76.7	Ref.	1.000

	CT	47	13.7	7	23.3	0.469	1.636 (0.431-6.208)
	CC	1	0.3	-	-	-	-
**rs10987759 dominant**	TT	294	86.0	23	76.7	Ref.	1.000
	CT+CC	48	14.0	7	23.3	1.000	120204327 (0.000-)
**rs10987759 recessive**	CC	1	0.3	-	-	Ref.	1.000
	CT+TT	341	99.7	30	100.0	1.000	223958208 (0.000-)

Table S169. Distribution of endoglin rs10987759 genotypes according to the presence of left ventricular hypertrophy (evaluated by Cornell voltage duration product)

SNP	Genotype	No		Yes		P value	OR (95% CI)
		N	%	N	%		
**rs10987759**	TT	297	85.1	20	87.0	Ref.	1.000
	CT	51	14.6	3	13.0	1.000	208430086 (0.000-)
	CC	1	0.3	-	-	-	-
**rs10987759 dominant**	TT	297	85.1	20	87.0	Ref.	1.000
	CT+CC	52	14.9	3	13.0	0.181	0.460 (0.147-1.437)
**rs10987759 recessive**	CC	1	0.3	-	-	Ref.	1.000
	CT+TT	348	99.7	23	100.0	0.553	0.676 (0.185-2.469)

Table S170. Distribution of endoglin rs10987759 genotypes according to the presence of left ventricular hypertrophy (evaluated by Sokolow-Lyon index)

SNP	Genotype	No		Yes		P value	OR (95% CI)
		N	%	N	%		
**rs10987759**	TT	317	85.0	4	80.0	Ref.	1.000
	CT	55	14.7	1	20.0	1.000	8578904.44 (0.000-)
	CC	1	0.3	-	-	-	-
**rs10987759 dominant**	TT	317	85.0	4	80.0	Ref.	1.000
	CT+CC	56	15.0	1	20.0	0.380	0.371 (0.040-3.397)
**rs10987759 recessive**	CC	1	0.3	-	-	Ref.	1.000
	CT+TT	372	99.7	5	100.0	0.997	1.008 (0.000-)

Table S171. Distribution of endoglin rs10987759 genotypes according to the presence of left ventricular hypertrophy (evaluated by Lewis index)

SNP	Genotype	No		Yes		P value	OR (95% CI)
		N	%	N	%		
**rs10987759**	TT	199	86.1	30	88.2	Ref.	1.000
	CT	31	13.4	4	11.8	0.519	0.641 (0.166-2.473)
	CC	1	0.4	-	-	-	-
**rs10987759 dominant**	TT	199	86.1	30	88.2	Ref.	1.000
	CT+CC	32	13.9	4	11.8	1.000	150279887 (0.000-)
**rs10987759 recessive**	CC	1	0.4	-	-	Ref.	1.000
	CT+TT	230	99.6	34	100.0	1.000	113440853 (0.000-)

Table S172. Distribution of endoglin rs10987759 genotypes according to the presence of abdominal obesity

SNP	Genotype	No		Yes		P value	OR (95% CI)
		N	%	N	%		
**rs10987759**	TT	151	85.8	171	84.2	Ref.	1.000
	CT	24	13.6	32	15.8	0.823	0.928 (0.484-1.780)
	CC	1	0.6	-	-	-	-
**rs10987759 dominant**	TT	151	85.8	171	84.2	Ref.	1.000
	CT+CC	25	14.2	32	15.8	1.000	2.752E+9 (0.000-)

rs10987759 recessive	CC	1	0.6	-	-	Ref.	1.000
	CT+TT	175	99.4	203	100.0	1.000	3.586E+9 (0.000-)

Table S173. Distribution of endoglin rs10987759 genotypes according to the presence of metabolic syndrome

SNP	Genotype	No		Yes		P value	OR (95% CI)
		N	%	N	%		
rs10987759	TT	247	86.6	75	79.8	Ref.	1.000
	CT	37	13.0	19	20.2	0.897	0.953 (0.461-1.970)
	CC	1	0.4	-	-	-	-
rs10987759 dominant	TT	247	86.7	75	79.8	Ref.	1.000
	CT+CC	38	13.3	19	20.2	1.000	240438355 (0.000-)
rs10987759 recessive	CC	1	0.4	-	-	Ref.	1.000
	CT+TT	284	99.6	94	100.0	1.000	361983296 (0.000-)

ANALYTICAL VALUES

Table S174. Distribution of endoglin rs10987759 genotypes according to the presence of elevated cholesterol plasma levels

SNP	Genotype	No		Yes		P value	OR (95% CI)
		N	%	N	%		
rs10987759	TT	94	82.4	217	86.1	Ref.	1.000
	CT	19	16.7	35	13.9	1.000	2.199E+9 (0.000-)
	CC	1	0.9	-	-	-	-
rs10987759 dominant	TT	94	82.5	217	86.1	Ref.	1.000
	CT+CC	20	17.5	35	13.9	1.000	3.898E+9 (0.000-)
rs10987759 recessive	CC	1	0.9	-	-	Ref.	1.000
	CT+TT	113	99.1	252	100.0	1.000	3.26E+9 (0.000-)

Table S175. Distribution of endoglin rs10987759 genotypes according to the presence of reduced cholesterol-high density lipoproteins plasma levels

SNP	Genotype	No		Yes		P value	OR (95% CI)
		N	%	N	%		
rs10987759	TT	232	85.0	79	84.9	Ref.	1.000
	CT	41	15.0	13	14.0	1.000	0.000 (0.000-)
	CC	-	-	1	1.1	-	-
rs10987759 dominant	TT	232	85.0	79	84.9	Ref.	1.000
	CT+CC	41	15.0	14	15.1	1.000	0.000 (0.000-)
rs10987759 recessive	CC	-	-	1	1.1	Ref.	1.000
	CT+TT	273	100.0	92	98.9	1.000	0.000 (0.000-)

Table S176. Distribution of endoglin rs10987759 genotypes according to the presence of elevated cholesterol-low density lipoproteins plasma levels

SNP	Genotype	No		Yes		P value	OR (95% CI)
		N	%	N	%		
rs10987759	TT	102	89.5	215	83.0	Ref.	1.000
	CT	12	10.5	43	16.6	0.344	1.397 (0.699-2.790)
	CC	-	-	1	0.4	-	-
rs10987759 dominant	TT	102	89.5	215	83.0	Ref.	1.000
	CT+CC	12	10.5	44	17.0	1.000	0.000 (0.000-)
rs10987759 recessive	CC	-	-	1	0.4	Ref.	1.000
	CT+TT	114	100.0	258	99.6	1.000	0.711 (0.000-)

Table S177. Distribution of endoglin rs10987759 genotypes according to the presence of elevated triglycerides plasma levels

SNP	Genotype	No		Yes		P value	OR (95% CI)
		N	%	N	%		
**rs10987759**	TT	241	85.1	81	84.4	Ref.	1.000
	CT	41	14.5	15	15.6	0.827	0.918 (0.428-1.973)
	CC	1	0.4	-	-	-	-
**rs10987759 dominant**	TT	241	85.2	81	84.4	Ref.	1.000
	CT+CC	42	14.8	15	15.6	0.978	1.009 (0.525-1.942)
**rs10987759 recessive**	CC	1	0.4	-	-	Ref.	1.000
	CT+TT	282	99.6	96	100.0	1.000	0.000 (0.000-)

Table S178. Distribution of endoglin rs10987759 genotypes according to the presence of dislipemia

SNP	Genotype	No		Yes		P value	OR (95% CI)
		N	%	N	%		
**rs10987759**	TT	20	90.9	301	84.5	Ref.	1.000
	CT	2	9.1	54	15.2	0.692	1.302 (0.352-4.816)
	CC	-	-	1	0.3	-	-
**rs10987759 dominant**	TT	20	90.9	301	84.5	Ref.	1.000
	CT+CC	2	9.1	55	15.4	1.000	0.000 (0.000-)
**rs10987759 recessive**	CC	-	-	1	0.3	Ref.	1.000
	CT+TT	22	100.0	355	99.7	1.000	0.000 (0.000-)

Table S179. Distribution of endoglin rs10987759 genotypes according to the presence of altered basal glycemia

SNP	Genotype	No		Yes		P value	OR (95% CI)
		N	%	N	%		
**rs10987759**	TT	282	84.4	40	88.9	Ref.	1.000
	CT	51	15.3	5	11.1	0.458	0.720 (0.303-1.712)
	CC	1	0.3	-	-	-	-
**rs10987759 dominant**	TT	282	84.4	40	88.9	Ref.	1.000
	CT+CC	52	15.6	5	11.1	1.000	257194035 (0.000-)
**rs10987759 recessive**	CC	1	0.3	-	-	Ref.	1.000
	CT+TT	333	99.7	45	100.0	1.000	185353933 (0.000-)

Table S180. Distribution of endoglin rs10987759 genotypes according to the presence of microalbuminuria

SNP	Genotype	No		Yes		P value	OR (95% CI)
		N	%	N	%		
**rs10987759**	TT	301	85.7	15	75.0	Ref.	1.000
	CT	49	14.0	5	25.0	0.116	0.412 (0.136-1.247)
	CC	1	0.3	-	-	-	-
**rs10987759 dominant**	TT	301	85.8	15	75.0	Ref.	1.000
	CT+CC	50	14.2	5	25.0	1.000	62332493.2 (0.000-)
**rs10987759 recessive**	CC	1	0.3	-	-	Ref.	1.000
	CT+TT	350	99.7	20	100.0	1.000	124355775 (0.000-)

CARDIOVASCULAR TESTS

Table S181. Distribution of endoglin rs10987759 genotypes according to the presence of elevated pulse pressure

SNP	Genotype	No		Yes		P value	OR (95% CI)
		N	%	N	%		
**rs10987759**	TT	238	82.9	84	91.3	Ref.	1.000
	CT	48	16.8	8	8.7	0.925	0.963 (0.443-2.094)
	CC	1	0.3	-	-	-	-
**rs10987759 dominant**	TT	238	82.9	84	91.3	Ref.	1.000
	CT+CC	49	17.1	8	8.7	1.000	490144871 (0.000-)
**rs10987759 recessive**	CC	1	0.3	-	-	Ref.	1.000
	CT+TT	286	99.7	92	100.0	1.000	237480278 (0.000-)

Table S182. Distribution of endoglin rs10987759 genotypes according to the presence of increased carotid intima-media thickness

SNP	Genotype	No		Yes		P value	OR (95% CI)
		N	%	N	%		
**rs10987759**	TT	263	84.8	55	86.6	Ref.	1.000
	CT	46	14.9	9	13.4	0.476	0.736 (0.316-1.712)
	CC	1	0.3	-	-	-	-
**rs10987759 dominant**	TT	263	84.8	58	86.6	Ref.	1.000
	CT+CC	47	15.2	9	13.4	1.000	229963818 (0.000-)
**rs10987759 recessive**	CC	1	0.3	-	-	Ref.	1.000
	CT+TT	309	99.7	67	100.0	1.000	202486168 (0.000-)

Table S183. Distribution of ALK1 rs10987759 genotypes according to the values of pulse wave velocity

SNP	Genotype	No		Yes		P value	OR (95% CI)
		N	%	N	%		
**rs10987759**	TT	266	83.9	51	89.5	Ref.	1.000
	CT	50	15.8	6	10.5	0.718	1.224 (0.408-3.671)
	CC	1	0.3	-	-	-	-
**rs10987759 dominant**	TT	266	83.9	51	89.5	Ref.	1.000
	CT+CC	51	16.1	6	10.5	1.000	131895052 (0.000-)
**rs10987759 recessive**	CC	1	0.3	-	-	Ref.	1.000
	CT+TT	316	99.7	57	100.0	1.000	106601613 (0.000-)

Table S184. Distribution of endoglin rs10987759 genotypes according to the presence of pathological ankle-brachial index

SNP	Genotype	Normal		Pathological		P value	OR (95% CI)
		N	%	N	%		
**rs10987759**	TT	318	85.0	3	75.0	Ref.	1.000
	CT	55	14.7	1	25.0	0.548	0.418 (0.024-7.183)
	CC	1	0.3	-	-	0.658	0.572 (0.048-6.805)
**rs10987759 dominant**	TT	318	85.0	3	75.0	Ref.	1.000
	CT+CC	56	15.0	1	25.0	1.000	0.725 (0.000-)
**rs10987759 recessive**	CC	1	0.3	-	-	Ref.	1.000
	CT+TT	373	99.7	4	100.0	1.000	1.335 (0.000-)

CURRENT DISEASES

Table S185. Distribution of endoglin rs10987759 genotypes according to the presence of cerebrovascular disease

SNP	Genotype	No		Yes		P value	OR (95% CI)
		N	%	N	%		
**rs10987759**	TT	320	85.1	2	66.7	Ref.	1.000
	CT	55	14.6	1	33.3	0.997	9959091.47 (0.000-)
	CC	1	0.3	-	-	-	-
**rs10987759 dominant**	TT	320	85.1	2	66.7	Ref.	1.000
	CT+CC	56	14.9	1	33.3	1.000	0.861 (0.000-)
**rs10987759 recessive**	CC	1	0.3	-	-	Ref.	1.000
	CT+TT	375	99.7	3	100.0	1.000	2.313 (0.000-)

Table S186. Distribution of endoglin rs10987759 genotypes according to the presence of cardiovascular disease

SNP	Genotype	No		Yes		P value	OR (95% CI)
		N	%	N	%		
**rs10987759**	TT	313	84.8	9	90.0	Ref.	1.000
	CT	55	14.9	1	10.0	0.843	1.189 (0.216-6.533)
	CC	1	0.3	-	-	-	-
**rs10987759 dominant**	TT	313	84.8	9	90.0	Ref.	1.000
	CT+CC	56	15.2	1	10.0	1.000	24067944.2 (0.000-)
**rs10987759 recessive**	CC	1	0.3	-	-	Ref.	1.000
	CT+TT	368	99.7	10	100.0	1.000	16194650.2 (0.000-)

Table S187. Distribution of endoglin rs10987759 genotypes according to the presence of kidney disease

SNP	Genotype	No		Yes		P value	OR (95% CI)
		N	%	N	%		
**rs10987759**	TT	312	85.0	4	100.0	Ref.	1.000
	CT	54	14.7	-	-	-	-
	CC	1	0.3	-	-	-	-
**rs10987759 dominant**	TT	312	85.0	4	100.0	Ref.	1.000
	CT+CC	55	15.0	-	-	-	-
**rs10987759 recessive**	CC	1	0.3	-	-	Ref.	1.000
	CT+TT	366	99.7	4	100.0	-	-

Table S188. Distribution of endoglin rs10987759 genotypes according to the presence of advanced retinopathy

SNP	Genotype	No		Yes		P value	OR (95% CI)
		N	%	N	%		
**rs10987759**	TT	11	86.0	25	75.8	Ref.	1.000
	CT	18	14.0	7	21.2	0.867	1.203 (0.138-10.512)
	CC	-	-	1	3.0	-	-
**rs10987759 dominant**	TT	111	86.0	7	87.5	Ref.	1.000
	CT+CC	18	14.0	1	12.5	0.867	1.203 (0.138-10.512)
**rs10987759 recessive**	CC	-	-	-	-	Ref.	1.000
	CT+TT	129	100.0	8	100.0	-	-

Table S189. Distribution of ALK1 rs3847859 genotypes according to the presence of controlled arterial pressure.

SNP	Genotype	< average		> average		P value	OR (95% CI)
		N	%	N	%		

**rs10987759**	TT	51	92.7	35	85.4	Ref.	1.000
	CT	4	7.3	6	14.6	0.223	0.432 (0.112-1.664)
	CC	-	-	-	-	-	-
**rs10987759 dominant**	TT	51	92.7	35	85.4	Ref.	1.000
	CT+CC	4	7.3	6	14.6	0.223	0.432 (0.112-1.664)
**rs10987759 recessive**	CC	-	-	-	-	Ref.	1.000
	CT+TT	55	100.0	41	100.0	-	-

Table S190. Distribution of endoglin rs10987759 genotypes according to the presence of target organ damage

**SNP**	**Genotype**	**No**		**Yes**		**P value**	**OR (95% CI)**
		N	%	N	%		
**rs10987759**	TT	54	90.0	30	88.2	Ref.	1.000
	CT	6	10.0	4	11.8	0.771	0.819 (0.213-3.141)
	CC	-	-	-	-	-	-
**rs10987759 dominant**	TT	54	90.0	30	88.2	Ref.	1.000
	CT+CC	6	10.0	4	11.8	0.771	0.819 (0.213-3.141)
**rs10987759 recessive**	CC	-	-	-	-	Ref.	1.000
	CT+TT	60	100.0	34	100.0	-	-

Table S191. Distribution of endoglin rs10987759 genotypes according to the presence of hypertension, diabetes and obesity

**SNP**	**Genotype**	**No**		**Yes**		**P value**	**OR (95% CI)**
		N	%	N	%		
**rs10987759**	TT	103	85.1	219	84.9	Ref.	1.000
	CT	18	14.9	38	14.7	0.362	1.374 (0.694-2.717)
	CC	-	-	1	0.4	-	-
**rs10987759 dominant**	TT	103	85.1	219	84.9	Ref.	1.000
	CT+CC	18	14.9	39	15.1	1.000	0.000 (0.000-)
**rs10987759 recessive**	CC	-	-	1	0.4	Ref.	1.000
	CT+TT	121	100.0	257	99.6	1.000	0.000 (0.000-)

Table S192. Distribution of endoglin rs10987759 genotypes according to the presence of hypertension and diabetes

**SNP**	**Genotype**	**No**		**Yes**		**P value**	**OR (95% CI)**
		N	%	N	%		
**rs10987759**	TT	136	86.1	186	84.1	Ref.	1.000
	CT	22	13.9	34	15.4	0.483	1.262 (0.658-2.422)
	CC	-	-	1	0.5	-	-
**rs10987759 dominant**	TT	136	86.1	186	84.2	Ref.	1.000
	CT+CC	22	13.9	35	15.8	1.000	0.000 (0.000-)
**rs10987759 recessive**	CC	-	-	1	0.5	Ref.	1.000
	CT+TT	158	100.0	220	99.5	1.000	0.000 (0.000-)

RISK SCALES

Table S193. Distribution of endoglin rs10987759 genotypes according to the presence of cardiovascular risk

**SNP**	**Genotype**	**Low**		**High**		**P value**	**OR (95% CI)**
		N	%	N	%		
**rs10987759**	TT	24	77.4	287	85.7	Ref.	1.000
	CT	7	22.6	47	14.0	1.000	1.328 (0.376-4.696)

	CC	-	-	1	0.3	-	-
**rs10987759 dominant**	TT	24	77.4	287	85.7	Ref.	1.000
	CT+CC	7	22.6	48	14.3	1.000	0.000 (0.000-)
**rs10987759 recessive**	CC	-	-	1	0.3	Ref.	1.000
	CT+TT	31	100.0	334	99.7	1.000	0.000 (0.000-)

Table S194. Distribution of endoglin rs10987759 genotypes according to the presence of diabetes (based on 2013 guidelines)

SNP	Genotype	No		Yes		P value	OR (95% CI)
		N	%	N	%		
**rs10987759**	TT	270	85.4	52	82.5	Ref.	1.000
	CT	46	14.6	10	15.9	0.928	1.038 (0.463-2.327)
	CC	-	-	1	1.6	-	-
**rs10987759 dominant**	TT	270	85.4	52	82.5	Ref.	1.000
	CT+CC	46	14.6	11	17.5	1.000	0.000 (0.000-)
**rs10987759 recessive**	CC	-	-	1	1.6	Ref.	1.000
	CT+TT	316	100.0	62	98.4	1.000	0.000 (0.000-)

Table S195. Distribution of endoglin rs10987759 genotypes according to the presence of hypertension (based on 2013 guidelines)

SNP	Genotype	No		Yes		P value	OR (95% CI)
		N	%	N	%		
**rs10987759**	TT	170	87.2	152	82.6	Ref.	1.000
	CT	24	12.3	32	17.4	0.486	1.257 (0.660-2.393)
	CC	1	0.5	-	-	-	-
**rs10987759 dominant**	TT	170	87.2	152	82.6	Ref.	1.000
	CT+CC	25	12.8	32	17.4	1.000	1.206E+9 (0.000-)
**rs10987759 recessive**	CC	1	0.5	-	-	Ref.	1.000
	CT+TT	194	99.5	184	100.0	1.000	1.777E+9 (0.000-)

RETINOPATHY

Table S196. Distribution of ALK1 rs10987759 genotypes according to the caliber of medium retinal arteries

SNP	Genotype	< average		> average		P value	OR (95% CI)
		N	%	N	%		
**rs10987759**	TT	100	80.0	104	92.0	Ref.	1.000
	CT	25	20.0	9	8.0	0.009	2.985 (1.318-6.764)
	CC	-	-	-	-	-	-
**rs10987759 dominant**	TT	100	80.0	104	92.0	Ref.	1.000
	CT+CC	25	20.0	9	8.0	0.009	2.985 (1.318-6.764)
**rs10987759 recessive**	CC	-	-	-	-	Ref.	1.000
	CT+TT	125	100.0	113	100.0	-	-

Table S197. Distribution of ALK1 rs10987759 genotypes according to the caliber of right retinal arteries

SNP	Genotype	< average		> average		P value	OR (95% CI)
		N	%	N	%		
**rs10987759**	TT	91	84.3	90	86.5	Ref.	1.000
	CT	17	15.7	14	13.5	0.608	1.225 (0.563-2.665)
	CC	-	-	-	-	-	-
**rs10987759 dominant**	TT	91	84.3	90	86.5	Ref.	1.000
	CT+CC	17	15.7	14	13.5	0.608	1.225 (0.563-2.665)

rs10987759 recessive	CC	-	-	-	-	Ref.	1.000
	CT+TT	108	100.0	104	100.0	-	-

Table S198. Distribution of ALK1 rs10987759 genotypes according to the caliber of minor retinal arteries

SNP	Genotype	< average		> average		P value	OR (95% CI)
		N	%	N	%		
rs10987759	TT	101	82.1	103	89.6	Ref.	1.000
	CT	22	17.9	12	10.4	0.072	2.020 (0.938-4.351)
	CC	-	-	-	-	-	-
rs10987759 dominant	TT	101	82.1	103	89.6	Ref.	1.000
	CT+CC	22	17.9	12	10.4	0.072	2.020 (0.938-4.351)
rs10987759 recessive	CC	-	-	-	-	Ref.	1.000
	CT+TT	123	100.0	115	100.0	-	-

Table S199. Distribution of ALK1 rs10987759 genotypes according to the caliber of left retinal arteries

SNP	Genotype	< average		> average		P value	OR (95% CI)
		N	%	N	%		
rs10987759	TT	86	82.7	96	89.7	Ref.	1.000
	CT	18	17.3	11	10.3	0.163	1.790 (0.790-4.058)
	CC	-	-	-	-	-	-
rs10987759 dominant	TT	86	82.7	96	89.7	Ref.	1.000
	CT+CC	18	17.3	11	10.3	0.163	1.790 (0.790-4.058)
rs10987759 recessive	CC	-	-	-	-	Ref.	1.000
	CT+TT	104	100.0	107	100.0	-	-

Table S200. Distribution of endoglin rs10987759 genotypes according to the values of left arteriovenous index (AVIx)

SNP	Genotype	< average		> average		P value	OR (95% CI)
		N	%	N	%		
rs10987759	TT	67	79.8	83	90.2	Ref.	1.000
	CT	17	20.2	9	9.8	0.044	2.494 (1.023-6.079)
	CC	-	-	-	-	-	-
rs10987759 dominant	TT	67	79.8	83	90.2	Ref.	1.000
	CT+CC	17	20.2	9	9.8	0.044	2.494 (1.023-6.079)
rs10987759 recessive	CC	-	-	-	-	Ref.	1.000
	CT+TT	84	100.0	92	100.0	-	-

Table S201. Distribution of endoglin rs10987759 genotypes according to the values of right arteriovenous index (AVIx)

SNP	Genotype	< average		> average		P value	OR (95% CI)
		N	%	N	%		
rs10987759	TT	68	82.9	70	88.6	Ref.	1.000
	CT	14	17.1	9	11.4	0.351	1.546 (0.618-3.866)
	CC	-	-	-	-	-	-
rs10987759 dominant	TT	68	82.9	70	88.6	Ref.	1.000
	CT+CC	14	17.1	9	11.4	0.351	1.546 (0.618-3.866)
rs10987759 recessive	CC	-	-	-	-	Ref.	1.000
	CT+TT	82	100.0	79	100.0	-	-

Table S202. Distribution of endoglin rs10987759 genotypes according to the values of medium arteriovenous index (AVIx)

SNP	Genotype	< average		> average		P value	OR (95% CI)
		N	%	N	%		
**rs10987759**	TT	99	80.5	103	91.2	Ref.	1.000
	CT	24	19.5	10	8.8	0.023	2.158 (1.138-5.571)
	CC	-	-	-	-	-	-
**rs10987759 dominant**	TT	99	80.5	103	91.2	Ref.	1.000
	CT+CC	24	19.5	10	8.8	0.023	2.158 (1.138-5.571)
**rs10987759 recessive**	CC	-	-	-	-	Ref.	1.000
	CT+TT	123	100.0	113	100.0	-	-

Table S203. Distribution of ALK1 rs10987759 genotypes according to the caliber of left retinal veins

SNP	Genotype	< average		> average		P value	OR (95% CI)
		N	%	N	%		
**rs10987759**	TT	103	89.6	79	82.3	Ref.	1.000
	CT	12	10.4	17	17.7	0.125	0.533 (0.239-1.190)
	CC	-	-	-	-	-	-
**rs10987759 dominant**	TT	103	89.6	79	82.3	Ref.	1.000
	CT+CC	12	10.4	17	17.7	0.125	0.533 (0.239-1.190)
**rs10987759 recessive**	CC	-	-	-	-	Ref.	1.000
	CT+TT	115	100.0	96	100.0	-	-

Table S204. Distribution of ALK1 rs10987759 genotypes according to the caliber of right retinal veins

SNP	Genotype	< average		> average		P value	OR (95% CI)
		N	%	N	%		
**rs10987759**	TT	98	86.7	83	83.8	Ref.	1.000
	CT	15	13.3	16	16.2	0.588	0.808 (0.373-1.751)
	CC	-	-	-	-	-	-
**rs10987759 dominant**	TT	98	86.7	83	83.8	Ref.	1.000
	CT+CC	15	13.3	16	16.2	0.588	0.808 (0.373-1.751)
**rs10987759 recessive**	CC	-	-	-	-	Ref.	1.000
	CT+TT	113	100.0	99	100.0	-	-

Table S205. Distribution of ALK1 rs10987759 genotypes according to the caliber of medium retinal veins

SNP	Genotype	< average		> average		P value	OR (95% CI)
		N	%	N	%		
**rs10987759**	TT	116	87.2	88	83.8	Ref.	1.000
	CT	17	12.8	17	16.2	0.503	0.779 (0.374-1.620)
	CC	-	-	-	-	-	-
**rs10987759 dominant**	TT	116	87.2	88	83.8	Ref.	1.000
	CT+CC	17	12.8	17	16.2	0.503	0.779 (0.374-1.620)
**rs10987759 recessive**	CC	-	-	-	-	Ref.	1.000
	CT+TT	133	100.0	105	100.0	-	-

Table S206. Distribution of ALK1 rs10987759 genotypes according to the caliber of major retinal veins

SNP	Genotype	< average		> average		P value	OR (95% CI)
		N	%	N	%		

**rs10987759**	TT	105	84.7	99	86.8	Ref.	1.000
	CT	19	15.3	15	13.2	0.697	1.158 (0.554-2.417)
	CC	-	-	-	-	-	-
**rs10987759 dominant**	TT	105	84.7	99	86.8	Ref.	1.000
	CT+CC	19	15.3	15	13.2	0.697	1.158 (0.554-2.417)
**rs10987759 recessive**	CC	-	-	-	-	Ref.	1.000
	CT+TT	124	100.0	114	100.0	-	-

KIDNEY DISEASE

Table S207. Distribution of endoglin rs10987759 genotypes according to the presence of pathological albumin/creatinine index

SNP	Genotype	Normal		Pathological		P value	OR (95% CI)
		N	%	N	%		
**rs10987759**	TT	20	16.1	135	78.9	Ref.	1.000
	CT	71	57.3	35	20.5	0.329	0.597 (0.211-1.684)
	CC	33	26.6	1	0.6	0.6	0.243 (0.081-0.733)
**rs10987759 dominant**	TT	20	16.1	135	78.9	Ref.	1.000
	CT+CC	104	83.9	36	21.1	1.000	65681543 (0.000-)
**rs10987759 recessive**	CC	33	26.6	1	0.6	Ref.	1.000
	CT+TT	91	73.4	170	99.4	1.000	0.000 (0.000-)

Table S208. Distribution of endoglin rs10987759 genotypes according to the presence of pathological kidney disease (evaluated by CKD-EPI)

SNP	Genotype	Normal		Pathological		P value	OR (95% CI)
		N	%	N	%		
**rs10987759**	TT	25	21.9	187	89.9	Ref.	1.000
	CT	46	40.4	21	10.1	0.674	1.159 (0.584-2.298)
	CC	43	37.7	-	-	0.614	1.178 (0.623-2.228)
**rs10987759 dominant**	TT	25	21.9	187	89.9	Ref.	1.000
	CT+CC	89	78.1	21	10.1	1.000	3.024E+9 (0.000-)
**rs10987759 recessive**	CC	43	37.7	-	-	Ref.	1.000
	CT+TT	71	62.3	208	100.0	1.000	1.334E+9 (0.000-)

HEART RATE

Table S209. Distribution of endoglin rs10987759 genotypes according to the values of heart rate (cutpoint: 100 bpm)

SNP	Genotype	< 100		> 100		P value	OR (95% CI)
		N	%	N	%		
**rs10987759**	TT	321	85.4	1	33.3	Ref.	1.000
	CT	54	14.3	2	66.7	0.571	0.446 (0.027-7.295)
	CC	1	0.3	-	-	0.364	0.275 (0.017-4.475)
**rs10987759 dominant**	TT	321	85.4	1	33.3	Ref.	1.000
	CT+CC	55	14.6	2	66.7	1.000	4171103.32 (0.000-)
**rs10987759 recessive**	CC	1	0.3	-	-	Ref.	1.000
	CT+TT	375	99.7	3	100.0	1.000	48157437.8 (0.000-)

Table S210. Distribution of endoglin rs10987759 genotypes according to the values of heart rate (cutpoint: 90 bpm)

SNP	Genotype	< 90	> 90	P value	OR (95% CI)

		N	%	N	%		
**rs10987759**	TT	314	85.1	8	80.0	Ref.	1.000
	CT	54	14.6	2	20.0	0.699	0.699 (0.113-4.313)
	CC	1	0.3	-	-	-	-
**rs10987759 dominant**	TT	314	85.1	8	80.0	Ref.	1.000
	CT+CC	55	14.9	2	20.0	1.000	44894175.1 (0.000-)
**rs10987759 recessive**	CC	1	0.3	-	-	Ref.	1.000
	CT+TT	368	99.7	10	100.0	1.000	66889650.6 (0.000-)

Table S211. Distribution of endoglin rs10987759 genotypes according to the values of heart rate (cutpoint: 70 bpm)

SNP	Genotype	< 70		> 70		P value	OR (95% CI)
		N	%	N	%		
**rs10987759**	TT	190	88.8	132	80.0	Ref.	1.000
	CT	24	11.2	32	19.4	0.002	0.362 (0.188-0.698)
	CC	-	-	1	0.6	-	-
**rs10987759 dominant**	TT	190	88.8	132	80.0	Ref.	1.000
	CT+CC	24	11.2	33	20.0	1.000	0.000 (0.000-)
**rs10987759 recessive**	CC	-	-	1	0.6	Ref.	1.000
	CT+TT	214	100.0	71	164	99.4	0.000 (0.000-)

Table S212. Distribution of endoglin rs10987759 genotypes according to the values of heart rate (cutpoint: 50 bpm)

SNP	Genotype	< 50		> 50		P value	OR (95% CI)
		N	%	N	%		
**rs10987759**	TT	3	75.0	319	85.1	Ref.	1.000
	CT	1	25.0	55	14.6	0.997	0.000 (0.000-)
	CC	-	-	1	0.3	-	-
**rs10987759 dominant**	TT	3	75.0	319	85.1	Ref.	1.000
	CT+CC	1	25.0	56	14.9	1.000	1.033 (0.000-)
**rs10987759 recessive**	CC	-	-	1	0.3	Ref.	1.000
	CT+TT	4	100.0	374	99.7	1.000	0.601 (0.000-)
